# The impact of autophagy modulation on phenotype and survival of cardiac stromal cells under metabolic stress

**DOI:** 10.1038/s41420-022-00924-7

**Published:** 2022-04-01

**Authors:** Isotta Chimenti, Vittorio Picchio, Francesca Pagano, Leonardo Schirone, Sonia Schiavon, Luca D’Ambrosio, Valentina Valenti, Maurizio Forte, Flavio di Nonno, Speranza Rubattu, Mariangela Peruzzi, Francesco Versaci, Ernesto Greco, Antonella Calogero, Elena De Falco, Giacomo Frati, Sebastiano Sciarretta

**Affiliations:** 1 Department of Medical Surgical Sciences and Biotechnologies, Sapienza University of Rome, Latina, Italy; 2 Mediterranea Cardiocentro, Napoli, Italy; 3 Biochemistry and Cellular Biology Istitute, CNR, Monterotondo, Italy; 4 Department of Clinical, Internal Medicine, Anaesthesiology and Cardiovascular Sciences, Sapienza University of Rome, Rome, Italy; 5 Haemodynamic and Cardiology Unit, “Santa Maria Goretti” Hospital, Latina, Italy; 6 IRCCS Neuromed, Pozzilli, Italy; 7 Department of Clinical and Molecular Medicine, Sapienza University of Rome, Rome, Italy; 8 Department of System Medicine, “Tor Vergata” University, Rome, Italy

**Keywords:** Mechanisms of disease, Macroautophagy, Translational research

## Abstract

Cardiac stromal cells (CSCs) embrace multiple phenotypes and are a contributory factor in tissue homeostasis and repair. They can be exploited as therapeutic mediators against cardiac fibrosis and remodeling, but their survival and cardioprotective properties can be decreased by microenvironmental cues. We evaluated the impact of autophagy modulation by different pharmacological/genetic approaches on the viability and phenotype of murine CSCs, which had been subjected to nutrient deprivation or hyperglycemia, in order to mimic relevant stress conditions and risk factors of cardiovascular diseases. Our results show that autophagy is activated in CSCs by nutrient deprivation, and that autophagy induction by trehalose or autophagy-related protein 7 (ATG7)-overexpression can significantly preserve CSC viability. Furthermore, autophagy induction is associated with a higher proportion of primitive, non-activated stem cell antigen 1 (Sca1)-positive cells, and with a reduced fibrotic fraction (positive for the discoidin domain-containing receptor 2, DDR2) in the CSC pool after nutrient deprivation. Hyperglycemia, on the other hand, is associated with reduced autophagic flux in CSCs, and with a significant reduction in primitive Sca1+ cells. Autophagy induction by adenoviral-mediated ATG7-overexpression maintains a cardioprotective, anti-inflammatory and pro-angiogenic paracrine profile of CSCs exposed to hyperglycemia for 1 week. Finally, autophagy induction by ATG7-overexpression during hyperglycemia can significantly preserve cell viability in CSCs, which were subsequently exposed to nutrient deprivation, reducing hyperglycemia-induced impairment of cell resistance to stress. In conclusion, our results show that autophagy stimulation preserves CSC viability and function in response to metabolic stressors, suggesting that it may boost the beneficial functions of CSCs in cardiac repair mechanisms.

## Introduction

Chronic maladaptive remodelling and progressive heart failure (HF) [1] represent life-threatening terminal conditions characterized by cardiomyocyte death, fibrosis, and remodelling [2, 3]. Nowadays, despite multiple advances in medical treatments and prognoses, valid options for end-stage HF patients are heart transplantation and left ventricular assist device implantation [4, 5]. However, these therapies are often limited by organ availability and several clinical complications [6]. Therefore, new therapeutic strategies against HF progression are needed in order to reduce its incidence and morbidity. The progression of the disease after acute myocardial infarction (MI) includes cardiomyocyte death by necrosis [7] and apoptosis [8], followed by scar formation caused by collagen-depositing interstitial cells to prevent heart rupture [9, 10]. Interstitial and stromal myocardial cells embrace a variety of phenotypes and functional states [11, 12], e.g. endothelial cells, fibroblasts and myofibroblasts [13, 14], telocytes [15], pericytes [16], mesenchymal-like cells [17], and immune cells [18], and play a primary role in cardiac homeostasis and repair after injury. Among resident stromal cells, a population of primitive cardiac stromal cells (CSCs) is discernible in the adult mammalian heart [19]. CSCs can be derived from myocardial tissue, and selected for a primitive non-activated phenotype by the spontaneous capacity to grow as 3D spheroids on a permissive substrate [20]. CSCs affect tissue architecture and the microenvironment [21], participate in tissue repair, and can be used as a therapeutic cell product [22, 23]. In fact, it was shown that in vivo transplantation of human primitive CSCs in the peri-infarct zone of a SCID-murine model of MI can have a positive effect on infarct size, cell death, and tissue revascularization [24]. This beneficial effect is largely due to paracrine mechanisms, i.e. release of diffusible molecules and vesicles with cardioprotective, pro-angiogenic, and anti-inflammatory properties [25–27]. These benefits on tissue repair, however, depend on specific features of CSCs. In fact, myocardial remodelling and cardiovascular risk factors, such as diabetes [28], may lead to pro-fibrotic polarization and loss of the reparative potential of CSCs [29, 30], with impaired paracrine potency [31–33]. Overall, the health status, comorbidities, and ongoing therapies of a patient can strongly affect the phenotype of CSCs [30–32, 34], and in this way their direct and indirect contribution to fibrosis and remodelling. Moreover, the survival of CSCs with a beneficial pro-repair phenotype after MI or during HF progression may be significantly affected by stress conditions, including nutrient deprivation in the ischemic and inflammatory milieu, or metabolic derangements [35].

For all the above reasons, CSCs can be considered targets for regenerative medicine approaches. Their survival in the damaged myocardium, as well as their reparative phenotype, represent important features to be considered and preserved for novel therapeutic strategies designed to counteract fibrosis and HF progression.

We hypothesized that, among other mechanisms, autophagy modulation may affect CSC survival and phenotype in response to stress. In fact, autophagy is a dynamic process involving degradation of damaged intracellular components and recycling of molecules useful for cell metabolism. Specific proteins, such as autophagy-related protein 7 (ATG7) and microtubule-associated proteins 1 A/1B light chain 3B (LC3-II), are involved in autophagy initiation, sustaining membrane nucleation and autophagosome formation [36]. It is noted that autophagy was reported to be dysregulated in patients with heart diseases or dysmetabolic conditions [37], while pharmacological activation of autophagy was shown to attenuate ischemic injury and postischemic chronic remodelling [38, 39]. Notwithstanding, the role of autophagy in CSC biology is still unclear.

The present study investigates the effects of autophagy modulation in CSCs when subjected to metabolic stress conditions, mimicking endogenous derangements associated with ischaemic injury (e.g. nutrient deprivation) and/or altered metabolism (e.g. hyperglycemia). We assayed CSCs for their viability and phenotype, and assessed whether autophagy activation can preserve a beneficial and protective paracrine profile in response to stress.

## Results

### CSC characterization

**Fig. 1:**
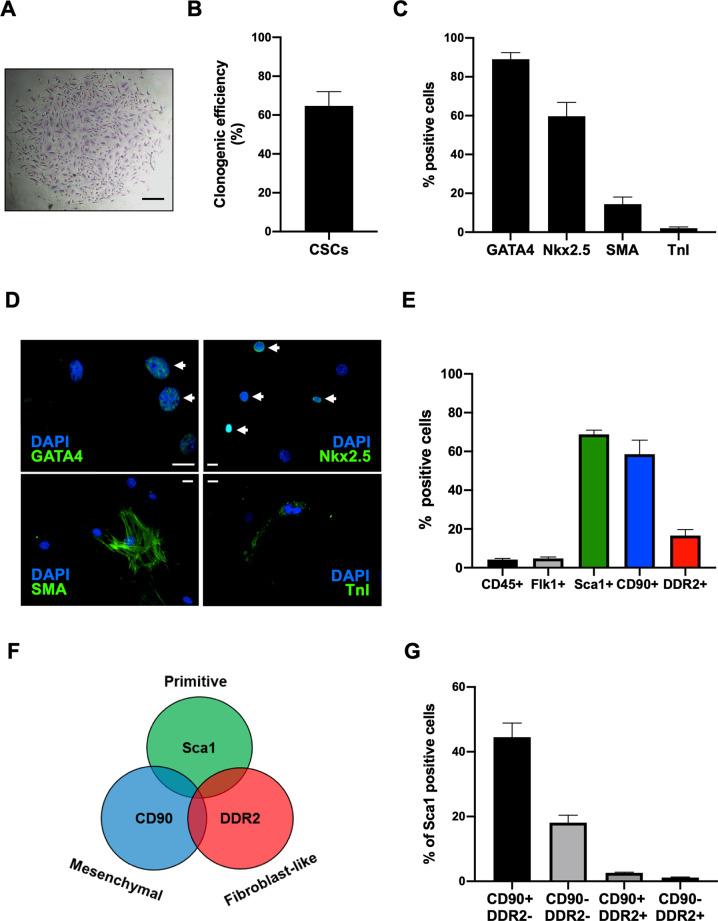
CSC characterization. **A** Representative microscopy image of a fixed GIEMSA-stained CSC colony. Scale bar=500 µm. **B** Quantification of clonogenic efficiency (*n*=6). **C** Quantification of positive cells (*n*=5), and representative immunofluorescence panels (**D**) for selected cardiac markers (GATA4, Nkx2.5, TnI, SMA). Arrows indicate positive nuclei. Scale bars=20 µm. **E** Quantification of single marker positivity by flow cytometry analysis of the whole CSC population (*n*=5). **F** Conceptual scheme of the association among the Sca1, CD90, and DDR2 markers with corresponding phenotypes. **G** Quantification by flow cytometry of the percentage of CD90 or DDR2 cells among the CD45-/Flk1-/Sca1+ population (*n*=3).

CSCs were isolated from primary explant cultures of C57BL6/J murine hearts, and selected through spontaneous spheroid formation for a primitive non-activated phenotype [40, 41]. To validate these features in our conditions, we tested isolated CSCs for clonogenic efficiency, which is a feature of undifferentiated phenotypes [40], and found a mean value of 64.7±7.3% (Fig. 1A, B). We also assessed their phenotype by immunofluorescence staining (Fig. 1C, D), and confirmed that 2.0±0.7% were positive for the differentiation marker troponin I (TnI) and 14.4±3.6% for smooth muscle actin (SMA) [42]. Moreover, cells were largely positive for the cardiac transcription factors GATA4 and Nkx2.5, in line with previous reports [43–45]. In addition, we characterized CSCs for surface marker expression using flow cytometry. We designed a panel to screen for the presence of contaminating haematopoietic or mature endothelial cells, and to better dissect possible phenotypic shifts in the CSC stromal population. Flow cytometry analysis at basal conditions (Fig. 1E, Supplementary fig. 1A) showed that isolated CSCs express very low levels of CD45 and Flk1 markers, consistent with a non-hematopoietic and non-endothelial lineage. Moreover, 66.7±2.2% of cells are positive for the primitive stromal marker stem cell antigen 1 (Sca1, which is associated with a non-activated primitive phenotype in situ) [12, 46], 56.7±8.9% are positive for the mesenchymal marker CD90, and 15.8±5.5 are positive for the mature/activated cardiac fibroblast marker ﻿discoidin domain-containing receptor 2 (DDR2) (Fig. 1F). In all the following experiments, we gated on the CD45-/Flk1- population (95.8±3.2% of total events), and used the DDR2 marker to quantify a differentiated myofibroblast population [47, 48]. Furthermore, analysis of the CD45-/Flk1-/Sca1+ population showed that in our conditions Sca1 and DDR2 are mutually exclusive (Fig. 1G, Supplementary Fig. 1B), and that 45.5±4.4% of the Sca1+ CSCs are also positive for CD90, consistent with a mesenchymal primitive phenotype (Sca+/CD90+/DDR2-).

### Autophagy is activated in CSCs in response to nutrient deprivation

**Fig. 2:**
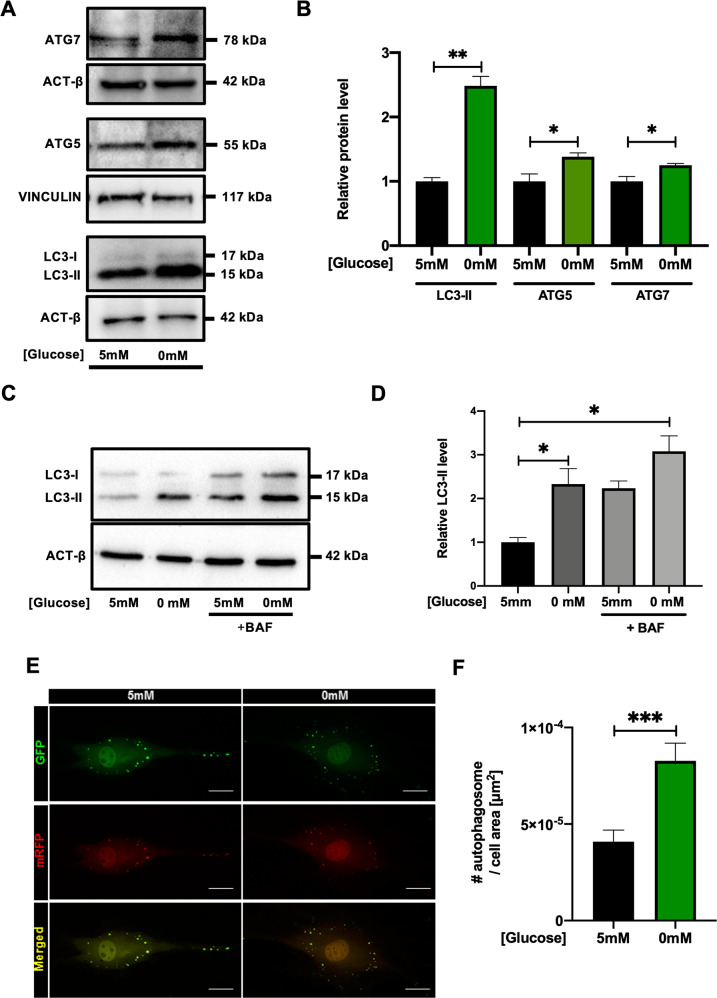
Autophagy is activated in CSCs in response to nutrient deprivation. Representative WB panels **(A)** of LC3-II, ATG5 and ATG7 protein levels, and corresponding densitometric analysis **(B)** after 4 hours of nutrient deprivation (0.1% FBS, 0 mM glucose) (*n*=3). Representative panels **(C)** and relative quantification of LC3-II **(D)** with or without concomitant treatment for 2 hours with 100 nM bafilomycin A1 (BAF) are also shown (*n*=3). **E** Representative fluorescence microscopy images of Ad-mRFP-GFP-LC3 transduced cells, exposed for 4 hours either to nutrient deprivation or control media (5 mM glucose), with corresponding quantification of the number of mRFP+/GFP+ autophagosomes per cell surface area **(F)**. Scale bars=20 µm. *P<0.05, **P<0.01, ***P<0.001.

Autophagy activation was confirmed in CSCs which were acutely subjected to 4 hours of nutrient deprivation (0.1% FBS, 0 mM glucose). Western blot analysis showed increased LC3-II, ATG5 and ATG7 protein levels under this stress condition, as compared to control (Fig. 2A, B). To define whether the observed increase in LC3-II protein levels was caused by increased autophagosome formation rather than decreased lysosome degradation, the lysosomal inhibitor bafilomycin A1 (BAF) was used. Treatment with 100 nM BAF showed LC3-II accumulation in cells which had been cultured both under normal conditions and in nutrient deprivation, with higher levels in starved cells (Fig. 2C, D). This result confirmed that the different levels of LC3-II protein were in fact due to higher autophagosome formation, and not to reduced degradation. Furthermore, autophagy activation in CSCs under nutrient deprivation was confirmed by ad-mRFP-GFP-LC3 transduction and fluorescent dots quantification, highlighting a significant increase in the number of autophagosomes, as compared to control (Fig. 2E, F).

### Autophagy stimulation reduces apoptosis in starved CSCs

**Fig. 3:**
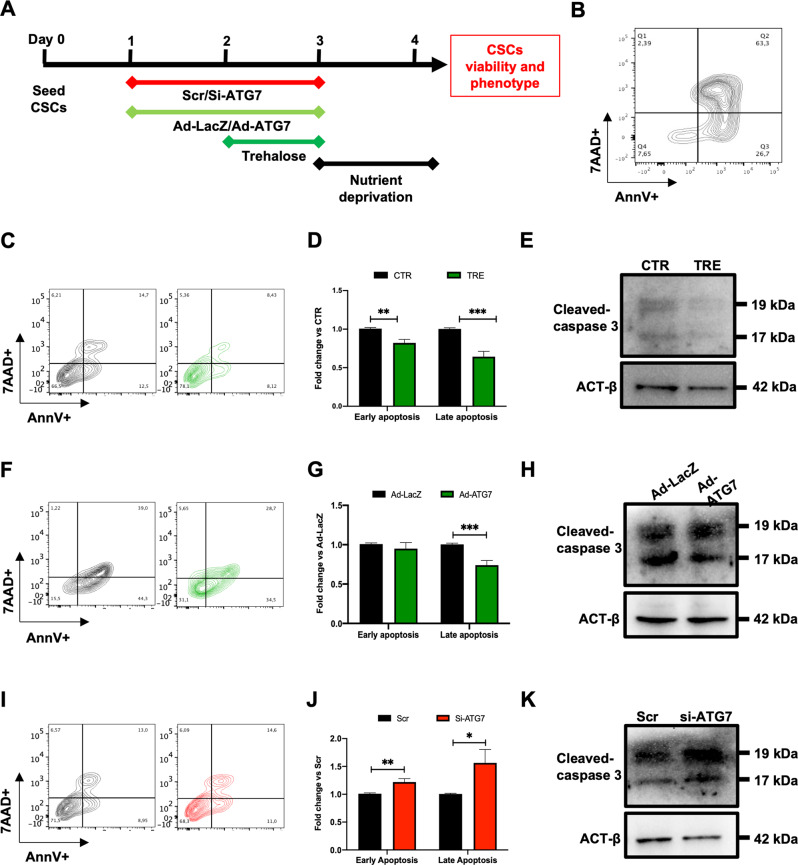
Autophagy stimulation reduces apoptosis in starved CSCs. **A** Experimental timeline and design for autophagy modulation and cell viability assessment. **B** Representative contour plot of the positive control of apoptotic induction. Representative flow cytometry density plots **(C)** and relative quantification **(D)** of early (Annexin-V+/7AAD-) and late (Annexin-V+/7AAD+) apoptotic CSCs after 32 hours of nutrient deprivation (0.1% FBS, 0 mM glucose), with or without pretreatment with 50 mM trehalose (TRE) for 24 hours (*n*=8); corresponding WB panel for cleaved caspase 3 is shown in paneI **(E)**. Representative flow cytometry density plots **(F)** and relative quantification **(G)** of early and late apoptotic cells after 32 hours of nutrient deprivation in CSCs that were previously transduced with ad-ATG7 or ad-LacZ 48 hours before stress (*n*=7); corresponding WB panel for cleaved caspase 3 is shown in panel **(H)**. Representative flow cytometry density plots **(I)** and relative quantification **(J)** of early and late apoptosis after 32 hours of nutrient deprivation in CSCs that were pre-treated with si-ATG7 or scramble control (Scr) transfection 48 hours before stress (*n*=7); corresponding WB panel for cleaved caspase 3 is shown in panel **(K)**. *P<0.05, **P<0.01, ***P<0.001.

We then investigated whether autophagy activation improves CSC survival in response to prolonged nutrient deprivation (up to 32 hours), which mimics the microenvironment features of the ischemic myocardium. Consolidated autophagy modulation tools were tested and optimized in our system. In particular, we either performed a 24 hour pretreatment with 50 mM of trehalose (TRE), a pharmacological autophagy activator, or overexpressed ATG7 (i.e. an initiator of macroautophagy) by adenovirus transduction (ad-ATG7) 48 hours before starvation to activate autophagy; in parallel we designed an RNA-interference approach to switch off autophagy through ATG7-knock down (Fig. 3A). We previously observed that 24 hours of TRE treatment significantly increases LC3-II protein levels, ATG7 mRNA expression, and the number of autophagosomes in CSCs, as compared with untreated controls (Supplementary Fig. 2). Similarly, both ATG7 mRNA and protein levels were significantly upregulated 48 hours after infection with ad-ATG7, as well as LC3-II protein, with respect to ad-LacZ control (Supplementary Fig. 3). Conversely, transfection of an ATG7-targeting small interference RNA (si-ATG7) significantly reduced ATG7 mRNA levels, and ATG7 and LC3-II protein levels after 48 hours, as compared with scramble (Scr) control (Supplementary fig. 4).

The autophagic response in CSCs was thus strengthened by either pretreatment with 50 mM of TRE or Ad-ATG7 transduction, before nutrient deprivation stress (32 hours, 0.1% FBS, 0 mM glucose) (Fig. 3A). Annexin-V/7AAD labelling by flow cytometry analysis was used to quantify early (Annexin-V+/7AAD-) and late (Annexin-V+/7AAD+) apoptotic cells, and caspase 3 cleavage was also assessed by WB. A positive apoptotic control sample was also considered (Fig. 3B). Flow cytometry analysis showed that TRE pre-treatment significantly reduced both early and late apoptosis after nutrient deprivation stress in CSCs (Fig. 3C, D). This effect was confirmed by decreased levels of caspase 3 cleavage (Fig. 3E). Similarly, ATG7 overexpression significantly reduced late apoptosis after stress (Fig. 3F, G), and reduced cleaved caspase 3 levels (Fig. 3H). Conversely, autophagy inhibition by si-ATG7 transfection significantly increased the proportion of both early and late apoptotic cells after 32 hours of nutrient deprivation, as compared with Scr control (Fig. 3I, J), with a consistent increase in caspase 3 cleavage (Fig. 3K).

### Autophagy stimulation enriches the primitive fraction of CSCs

**Fig. 4:**
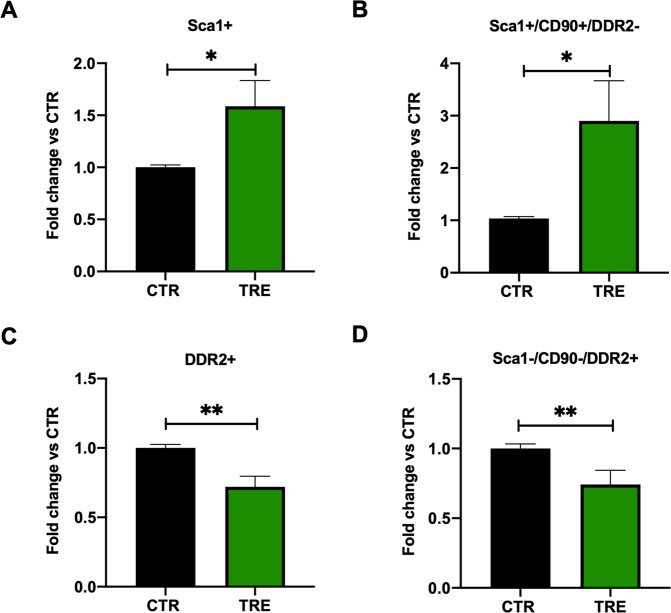
Autophagy stimulation enriches CSCs with primitive mesenchymal cells. Normalized quantification by flow cytometry of the CD45-/Flk1-/Sca1+ population **(A)**, the Sca1+/CD90+/DDR2- mesenchymal primitive subpopulation **(B)**, the whole CD45-/Flk1-/DDR2+ fibroblast-like fraction **(C)**, and of the Sca1-/CD90-/DDR2+ subpopulation **(D)** in CSCs after 32 hours of nutrient deprivation stress, with or without pretreatment with 50 mM trehalose (TRE) for 24 hours (*n*=8). *P<0.05, **P<0.01.

We then checked whether autophagy stimulation affects the relative cell-type composition within the CSC population by the use of flow cytometry to evaluate the surface marker expression (Fig. 1E, F). While Ad-ATG7 transduction and si-ATG7 transfection did not modulate CSC immunophenotype after 32 hours of nutrient deprivation (Supplementary Fig. 5), TRE pre-treatment, on the other hand, preserved a significantly higher proportion of Sca1+ cells after stress, including the Sca1+/CD90+/DDR2- primitive mesenchymal fraction (Fig. 4A, B). By contrast, the proportion of DDR2+ fibroblast-like cells (including the Sca1-/CD90-/DDR2+ subpopulation) was significantly reduced after nutrient deprivation stress in TRE-treated CSCs, as compared to controls (Fig. 4C, D). These results, together with the above-mentioned outcome on cell viability, suggest that TRE may be more effective than ad-ATG7 in activating autophagy in CSCs under our conditions, albeit a direct comparison is not feasible given the different nature of the two stimuli.

### Autophagy is reduced in CSCs exposed to hyperglycemia

**Fig. 5:**
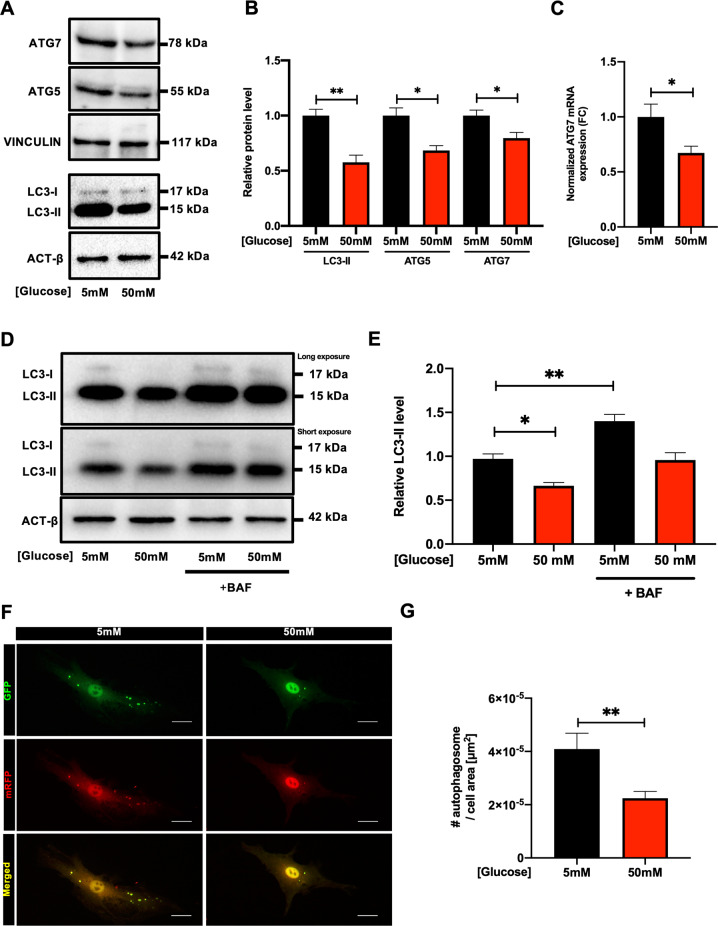
Autophagy is reduced in CSCs exposed to high-glucose concentration. Representative WB panels **(A)** and relative densitometric quantification **(B)** of LC3-II (*n*=8), ATG5, and ATG7 protein expression after exposure for 24 h to 5 mM or 50 mM glucose (*n*=3). **C** ATG7 mRNA expression levels after 24 h of culture in 5 mM or 50 mM glucose (n=5). Representative WB panels **(D)** and relative densitometric quantification **(E)** of LC3-II expression profile after 24 h of exposure to 50 mM glucose, with or without treatment for 2 hours with 100 nM bafilomycin A1 (BAF) (*n*=4). LC3-I and II panel is displayed twice with different exposure times, for the optimized detection of the two bands (higher exposure, top panel; lower exposure, lower panel). **F** Representative fluorescence microscopy images of Ad-mRFP-GFP-LC3 transduced cells, exposed for 24 h to 50 mM glucose or 5 mM glucose as control. **G** Corresponding quantification of the number of mRFP+/GFP+ autophagosomes per cell surface area in 50 mM glucose or 5 mM control (*n*=3). Scale bars=20 µm. *P<0.05, **P<0.01.

CSC autophagy, viability, and immunophenotype were then investigated after exposure to high glucose (50 mM) to mirror the clinically detrimental condition of hyperglycemia. This latter is associated with several cardiovascular risk conditions and comorbidities (e.g. metabolic syndrome, diabetes), and it is known to impair autophagy [49]. CSCs were cultured in 50 mM of glucose for 24 hours. We found that LC3-II, ATG5 and ATG7 protein levels were significantly reduced in hyperglycemic conditions (Fig. 5A, B), as well as ATG7 mRNA expression (Fig. 5C). To demonstrate that the downregulation of LC3-II protein was caused by a decrease in autophagosome formation rather than increased autophagic flux, BAF treatment was performed in addition to high-glucose stress. We observed that hyperglycemia reduced LC3-II level both in the presence or the absence of BAF treatment (Fig. 5D, E). Autophagy reduction by hyperglycemia was also confirmed by ad-mRFP-GFP-LC3 transduction and fluorescent dots quantification, highlighting a significant reduction in the number of autophagosomes in CSCs exposed to high glucose versus control (Fig. 5F, G). CSC viability (evaluated by Annexin V/7AAD labelling) was not affected by hyperglycemia, up to one week (Supplementary Fig. 6A).

### Hyperglycemia deprives CSCs of the primitive fraction

**Fig. 6:**
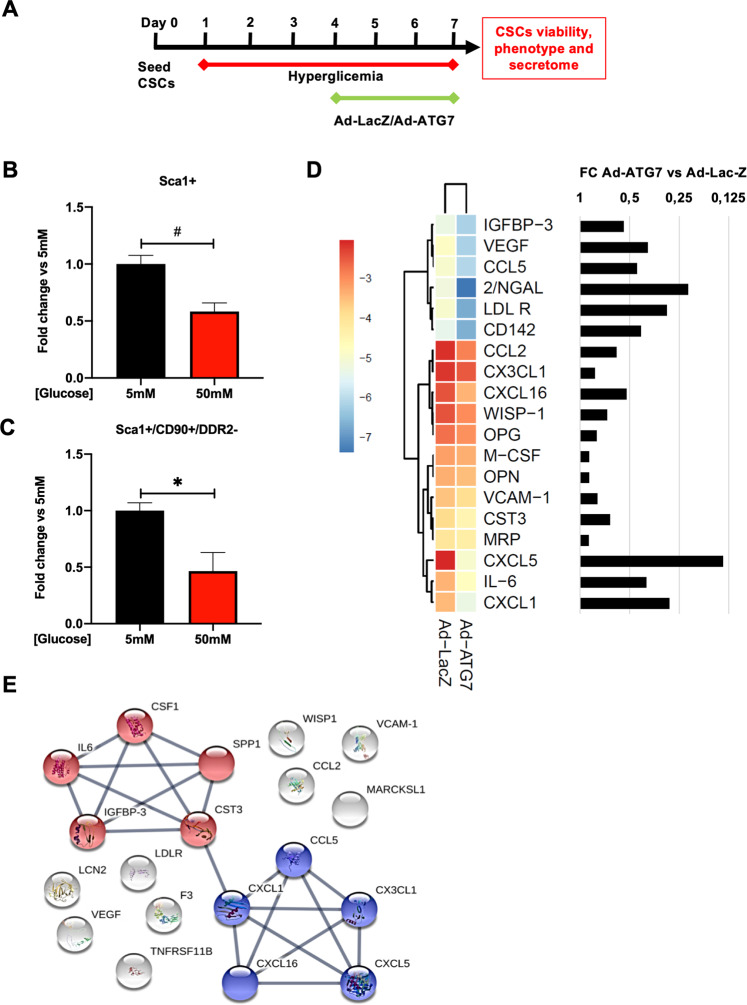
Hyperglycemia deprives CSCs of primitive cells and alters their paracrine profile. **A** Experimental timeline and design for autophagy modulation during hyperglycemia treatment. Quantification by flow cytometry of total Sca1+ **(B)** and Sca1+/CD90+/DDR2- **(C)** cells in the CSC population after 1 week of culture in 50 mM glucose, or 5 mM as control (n=3). **D** Heatmap with hierarchical clustering of Euclidean distance, calculated on Log2 values from normalized optical density of protein arrays on conditioned media from ad-ATG7 or ad-LacZ transduced CSCs exposed to 50 mM glucose; the heatmap is shown associated to the fold change (FC) histogram of ad-ATG7 versus ad-LacZ levels, for a selection of the most modulated cytokines. **E** Network of functional associations from the STRING database among cytokines of interest, showing only associations with edge confidence >0.7 (high/highest). *P<0.05, #P<0.0001.

We screened whether hyperglycemia may polarize CSC phenotype towards fibrotic features over time by flow cytometry analysis. We found a significant change in the surface marker profile of CSCs after 1 week of culture in 50 mM glucose (Fig. 6A). In actual fact, we observed that the Sca1+ population was significantly reduced in CSCs after 7 days of hyperglycemia, with respect to normoglycemia (Fig. 6B), as well as the Sca1+/CD90+/DDR2- fraction (Fig. 6C). However, no significant modulation in the proportion of DDR2+ cells could be detected in these conditions. In order to test whether autophagy reactivation could oppose the depletion of Sca1+ cells during high glucose stress, CSCs were transduced with Ad-ATG7 at day 4 of hyperglycemia treatment. Then they were analyzed by flow cytometry analysis at the end of a 1 week-treatment (Fig. 6A). Late autophagy reactivation by ATG7 overexpression did not exert any detectable effect on CSC surface marker profile during prolonged culture in high glucose conditions (Supplementary fig. 6B-E).

### Autophagy counteracts the detrimental paracrine profile of CSCs after hyperglycemia

CSCs act significantly through paracrine mechanisms, so we investigated whether their paracrine profile was different after one week of exposure to hyperglycemia. Since this was a non-lethal stress with no effect on the proportion of Annexin V/7AAD labelled cells (Supplementary fig. 6A), we could exclude any altered cytokine release due to cell death. We screened conditioned media by protein arrays, and then analyzed the transformed optical density data of each cytokine by hierarchical clustering and heatmap, and by calculating the ad-ATG7/ad-LacZ ratio. We detected a consistent decrease in the levels of several proinflammatory cytokines and chemokines in CSC-conditioned media after autophagy activation by Ad-ATG7 transduction, as compared to ad-LacZ control (Fig. 6D). In particular, we found a consistent decrease in the secreted levels of chemokines CXCL1, CXCL5, CXCL16, CCL5, CCL2, and CXCL3, as well as other cytokines such as IL-6 and colony-stimulating factor 1 (CSF1), in ATG7-overexpressing CSCs after hyperglycemic stress, as compared to LacZ-overexpressing control cells. It is interesting to note that we also detected a downregulation of Lipocalin-2 (NGAL), VEGF, IGF-binding protein 3 (IGFBP3), and low-density lipoprotein receptor (LDLR) in CSCs with ATG7 overexpression in response to hyperglycemia treatment. Functional association network analysis on the STRING database demonstrated a significant enrichment (False Discovery Rate >0.05) in several GO terms related to biological processes, molecular functions, and cellular components in ATG7-overexpressing CSCs versus control cells in response to hyperglycemia (Supplementary Table 1). Specifically, two functional sub-networks emerged, as shown in Fig. 6E: one among chemokines (except CCL2), all belonging to the “Chemokine receptors bind chemokines” pathway (Supplementary fig. 7A); another one connecting IL-6, IGFBP3, CSF1, cystatin C (CST3), and secreted phosphoprotein 1 (SPP1), sharing the “regulation of IGF activity by IGFBP” pathway (Supplementary Fig. 7B).

**Fig. 7:**
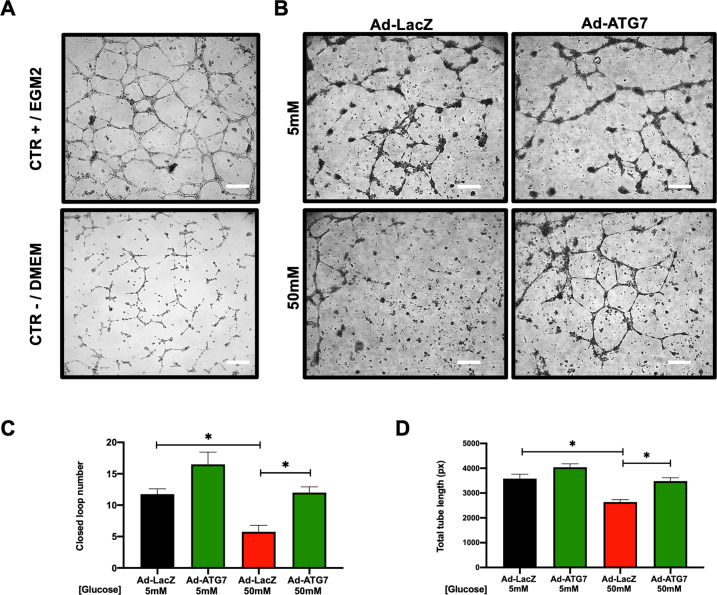
Autophagy induction enhances the pro-angiogenic paracrine capacity of CSCs. Tube-forming assay was performed with HUVECs exposed to conditioned media from ad-ATG7 or ad-LacZ transduced CSCs, in 5 or 50 mM glucose. Representative culture images are shown (**A**, **B**), together with the quantification of closed-loop number **(C)** and total tube length **(D)** (*n*=4). Scale bars=200 µm. *P<0.05.

In order to assess the functional potential of this altered paracrine profile as a possible microenvironmental cue, we tested the ability of CSC-conditioned media to affect endothelial tube formation. Complete endothelial growth medium (EGM) was used as the positive control, while basal DMEM medium 0.1% FBS was considered as the negative control (Fig. 7A). Human umbilical vein endothelial cells (HUVECs) were exposed to conditioned media from ad-ATG7-transduced CSCs in hyperglycemia, and they were able to form significantly more closed loops (Fig. 7B, C) with a significantly higher total tube length (Fig. B, D), as compared to ad-LacZ-transduced CSCs.

Overall, autophagy reactivation was capable of promoting a less inflammatory/fibrotic and a more angiogenic/cardioprotective paracrine profile.

### Autophagy induction protects CSCs from hyperglycemia-impaired stress resistance

**Fig. 8:**
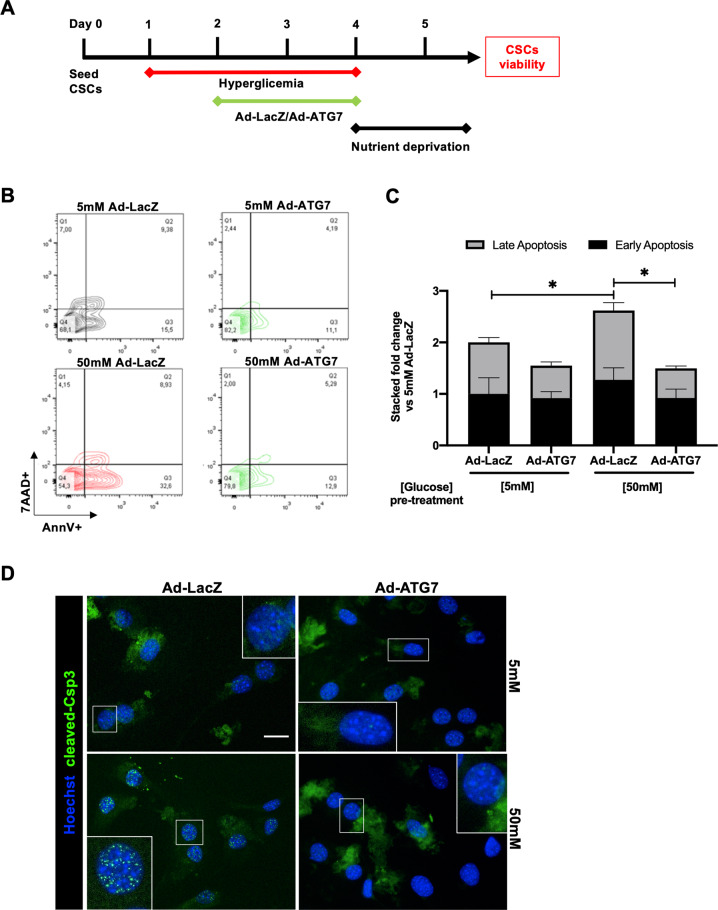
Autophagy induction protects CSCs from hyperglycemia-impaired stress resistance. **A** Experimental timeline of hyperglycemia (50 mM) pretreatment for 72 hours, with parallel ad-ATG7/ad-LacZ transduction for autophagy modulation, followed by 32 hours of nutrient deprivation stress (0.1% FBS, 0 mM glucose). Representative flow cytometry density plots **(B)** and relative quantification **(C)** of early (Annexin-V+/7AAD-) and late (Annexin-V+/7AAD+) apoptotic CSCs, to evaluate hyperglycemia-impaired and autophagy-enhanced resistance to nutrient deprivation stress (*n*=4). Representative immunofluorescence panels are displayed with higher magnification insets **(D)**, showing differential nuclear translocation of cleaved caspase 3 (Csp3) in the different conditions. *P<0.05. Scale bar = 20 µm.

Overall, hyperglycemia was able to impair autophagy activation and to reduce the primitive fraction in CSCs. Hyperglycemia is also known to reduce cardiac cell resistance to stress, such as ischemia and nutrient deprivation [50, 51]. Therefore, we investigated whether exposure to high glucose levels may affect CSC resistance to nutrient deprivation and, if so, whether autophagy reactivation may be a protective factor. CSCs were infected with ad-ATG7 or ad-LacZ, and concomitantly exposed to 50 mM glucose for 3 days, followed by 32 hours of nutrient deprivation stress (0.1% FBS, 0 mM glucose) (Fig. 8A). Viability analysis showed that hyperglycemia pre-conditioning determined an increase in apoptotic cells detected by AnnexinV/7AAD labelling after nutrient deprivation in ad-LacZ transduced control CSCs. Conversely, cell sensitivity to nutrient deprivation stress after exposure to high glucose was significantly rescued by ATG7-overexpression, as shown by representative density plots and relative quantification (Fig. 8B, C), and by nuclear translocation of cleaved caspase 3 (Fig. 8D) [52].

## Discussion

Primitive mesenchymal-like CSCs can be identified and isolated from the adult mammalian heart by different criteria and protocols [24, 32, 46, 53]. These cells are identified as non-activated stromal cells in myocardial homeostasis [12, 21], and can also be utilised for regenerative medicine strategies [54]. In fact, previous studies showed that CSCs can exert beneficial effects in the injured myocardium, mostly by positive microenvironmental conditioning through paracrine factors, which are able to render activated fibroblasts anti-fibrotic, and to polarize macrophages towards M2-like anti-inflammatory features [24, 26, 55–58]. Moreover, CSC phenotype can shift from cardioprotective features towards a pro-inflammatory and pro-fibrotic behaviour in response to different cues [29, 33, 59, 60]. This phenotypic shift within the heterogeneous stromal population may significantly affect adaptation and therapeutic mechanisms in response to stress and injury [33]. Therefore, CSC survival and phenotype balance in the injured and remodelled myocardium represent important features to be considered and studied, as a potential therapeutic target for anti-fibrotic or regenerative strategies [61, 62]. We did, in fact, aim to determine whether a cytoprotective strategy through autophagy enhancement could exert a positive effect on survival and phenotype of CSCs in response to different kinds of metabolic stress.

Our results show increased activation of autophagy in CSCs exposed to nutrient deprivation, in line with the known protective role of this degradation system in response to energy stress [63, 64] or oxidative stress [65]. Autophagy boosting with both ATG7-overexpression and TRE treatment before starvation induced a significant increase in CSC survival. Conversely, ATG7 downregulation exacerbated cell death. These data confirm the concept that autophagy can increase the resilience of cardiac cells (now including CSCs) towards nutrient deprivation in ischemic settings.

We also described the relative abundance of stromal cells with a primitive phenotype versus an activated/fibrotic phenotype within the CSC population, designing an immunophenotyping strategy based on the wide experience available from the literature from several groups [12, 66]. It is worth arguing that, despite the ongoing debate on the complex phenotypic profile of cardiac fibroblasts and CSCs [67], DDR2 is a protein expressed in mature cardiac fibroblasts and directly involved in their functions [47, 48, 68]. We therefore considered it as a positive marker for the presence of activated functional fibroblasts in culture, regardless of the concomitant staining for both CD90 and Sca1. Overall, we observed a shift towards a pro-fibrotic phenotype of surviving CSCs after exposure to FBS and glucose deprivation, that was significantly counteracted by autophagy boosting. In fact, CSCs that were pre-treated with TRE showed a higher relative amount of total Sca1+ cells, including Sca1+/CD90+/DDR2- mesenchymal primitive cells, and a reduced proportion of cells expressing the fibroblast marker DDR2. This finding is in line with the notion that fibrotic tissues display decreased autophagy [69], and that autophagy homeostasis is crucial to preserving an undifferentiated primitive phenotype [70].

Among the main cardiovascular risk factors and comorbidities, glucose dysmetabolism represents a specific pathogenetic factor in cardiomyopathy development [28]. The consequent detrimental microenvironment may impair the reparative capacity of CSCs in the damaged myocardium [31]. Here we investigated whether hyperglycemia, which is associated with metabolic syndrome and diabetes, may cause impairment of the phenotype of CSCs, and of their resistance to nutrient deprivation stress. Indeed, we showed that acute exposure to hyperglycemia reduces the autophagic flux in CSCs, in line with several other studies about autophagy in diabetic cardiomyopathy [37]. Moreover, one week of hyperglycemia significantly depleted the primitive pool of CSCs, although delayed autophagy enhancement could not counteract this phenotypic shift in our conditions. Nonetheless, after intermediate autophagy activation during prolonged hyperglycemic stress, CSCs released lower levels of several chemokines and cytokines associated with inflammation and immune cell recruitment [71]. These results are consistent with the available data regarding the direct effects of autophagy activation on reducing transcription, processing, and secretion of numerous pro-inflammatory cytokines [72, 73]. Functional association analyses in the STRING database included all downregulated chemokines in the same sub-network, indicating overall reduced pro-inflammatory signalling in ATG7-overexpressing CSCs. Moreover, several other modulated cytokines were included and interlinked in another sub-network, as they were united by functional association with the “regulation of IGF activity by IGFBP” pathway. The IGF-1 pathway is indeed involved in cardioprotective and adaptive mechanisms in response to metabolic stress and ischemia [74]. Interestingly, IGF-1 bioavailability has been shown to be tightly regulated in CSCs cultured in a 3D microenvironment [75], and to mediate their cardioprotective features [76]. Interestingly, we also found reduced secreted levels of Lipocalin-2 (NGAL), that is known to positively correlate with HF progression. In fact, NGAL activation can suppress the beneficial cardiac autophagic response to ischemia, contributing to enhanced ischemia-induced cell death and cardiac dysfunction [77]. Finally, autophagy activation in CSCs exposed to hyperglycemia consistently reduced VEGF release, as described in other cell types [78]. Finally, we showed that autophagy boosting could preserve the paracrine capacity of CSCs to support the angiogenic function of endothelial cells under hyperglycemic conditions, indicating positive microenvironmental conditioning by CSCs upon enhanced autophagy activation.

We also investigated if autophagy could improve cell resistance to stress after exposure to high glucose, that could represent a CSC-independent preconditioning strategy for the myocardial microenvironment in vivo [49]. Indeed, our results showed that autophagy activation during hyperglycemia treatment could significantly reduce cell death in CSCs subsequently exposed to nutrient deprivation. Overall, these results suggest that autophagy stimulation after initial exposure to high glucose, albeit not having the ability to counteract the depletion in the primitive fraction of CSCs, may at least reduce their pro-inflammatory signalling while enhancing pro-angiogenic signals in pathological conditions, such as diabetes or metabolic syndrome. Moreover, TRE- or ATG7-driven activation of autophagy may be considered as a strategy for empowering transplanted primitive CSCs in the injured and/or dysmetabolic myocardium, enhancing both their survival and paracrine potency, thus possibly ameliorating myocardial regeneration. Future studies are required to address this issue.

In conclusion, our results show that autophagy stimulation may prove useful for CSC survival and for preservation of a positive cardio-protective phenotype, in particular when exposed to dysmetabolic stressors. Finally, autophagy promotion may potentially increase the efficacy of cardiac cell therapy protocols by improving cell resistance to stress after transplantation in the damaged tissue, and by preserving a beneficial paracrine phenotype of engrafted cells.

## Material and methods

### Cardiac stromal cell culture

Cardiac stromal cells (CSCs) were isolated as cardiosphere-derived cells, as previously described as a resident cell population of non-hematopoietic stromal cells containing primitive undifferentiated cells [79, 80] . CSCs were derived from atrial tissue of 4-week old C57Bl6J mice. In brief, atrial tissue was fragmented, digested, and plated as explant cultures in Petri dishes previously coated with Fibronectin (FN) (CELL guidance system, Cambridge, UK) in complete explant media (CEM): Iscove’s modified Dulbecco’s medium (IMDM) (Sigma-Aldrich, St Louis, USA) supplemented with 20% FBS (Sigma-Aldrich), 1% penicillin-streptomycin (Sigma-Aldrich), 1% L-glutamine (Lonza), and 0.1 mM 2-mercaptoethanol (Thermo-Fisher, Waltham, USA). Explant-derived cells were collected after 3 weeks by sequential washes with Ca^2+^-Mg^2+^ free PBS, 0.48 mM/L Versene (Thermo-Fisher) for 3 minutes, and with 0.05% trypsin-EDTA (Lonza, Basel, CH) for 5 minutes at room temperature under visual control. Collected cells were seeded at low density (2,5x10^4^ cells/cm^2^) in 12-well plate previously coated with poly-D-lysine (Corning, New York, USA) in spheroid-growing medium: 35% complete IMDM/65% DMEM–F-12 mix, containing 2% B27, 0.1 mmol/L 2-mercaptoethanol, 10 ng/mL epidermal growth factor (EGF; Peprotech, London, UK), 20 ng/mL fibroblast growth factor (FGF), 40 nmol/L cardiotrophin-1 (both Miltenyi Biotec, Bergisch Gladbach, DU), 40 nmol/L thrombin (Sigma-Aldrich), antibiotics and L-Glu as in CEM. After 1 week, cells spontaneously formed 3D-spheroids, which were collected by gently pipetting without enzymatic digestion, and then expanded as a monolayer on FN-coating in CEM. For these experiments cells were plated at a density of 1.25×10^4^ cells/cm^2^. Eight CSCs lines were used, each obtained from a tissue pool of five mice.

### Colony-forming unit assay

The colony-forming unit (CFU) assay was performed as previously described [81]. Briefly, CSCs were seeded at low density (10 cells/cm^2^) in CEM on FN-coated plates, and incubated for 14 days at 37 °C with 5% CO_2_. Colonies were then fixed with 4% paraformaldehyde, stained with Giemsa (Sigma-Aldrich) for 1 hour, and counted under an optical microscope. A cluster with at least 50 cells was considered to be a colony; clonogenic efficiency was calculated as number of colonies per plated cells, and plotted as percentage.

### Immunostaining and fluorescence microscopy analysis

For immunofluorescence, CSCs cultured for 1 day in 3% FBS-CEM on fibronectin-coated plates were fixed for 10 minutes with 4% paraformaldehyde at 4 °C, and permeabilized with 0.1% Triton X-100 (Sigma-Aldrich) in PBS with 1% BSA. Nonspecific antibody binding sites were blocked with 10% goat serum (Sigma-Aldrich) in PBS, before overnight incubation at 4 °C with primary antibodies: GATA-4 (ab84593), NKX2-5 (ab97355), SMA (ab5694) (all Abcam, Cambridge, UK), TnI (sc-365446, Santa Cruz Biotechnology, Dallas, USA), cleaved caspase 3 (9661 s, Cell Signalling Technologies, Danvers, USA). After thorough washing, slides were incubated for 2 hours at room temperature with the appropriate Alexa-conjugated secondary antibodies (A11029, A1103, Thermo-Fisher) and DAPI nuclear dye (D1306, Thermo-Fisher). Slides were mounted in 70% glycerol in PBS. Image capture was performed on a Nikon Eclipse Ni microscope equipped with VICO system and NIS-Elements AR 4.30.02 software with a 20X objective (Nikon Corporation, Tokyo, JA). Percentage of positive cells was calculated by semi-automatic counting normalized to the number of nuclei per each 20X field.

### Western blot

Total protein cell extracts were made using a Lemli 2x with 5% β-mercapto-ethanol buffer, and stored at −80 °C until western blotting analysis. Equal volumes of protein lysate were boiled at 100 °C for 5 minutes, loaded on sodium dodecyl sulfate 15% polyacrylamide gels for electrophoresis (SDS-PAGE), and transferred to PVDF membranes (Sigma-Aldrich). Membranes were then blocked with 3% BSA for 1 hour at room temperature, and incubated with primary antibodies against tubulin (2148 S, Cell Signalling Technologies), vinculin (sc-73264, Santa Cruz), β-actin (C54070S, Cell Signalling Technologies), ATG7 (8558 S, Cell Signalling Technologies), cleaved caspase 3 (9661 s, Cell Signalling Technologies), ATG5 (NB110–53818, Novus Biological), and LC3 (M186–3, MBL International, Woburn, USA) at 4 °C overnight under gentle agitation. Membranes were then washed three times with TBS-0.01% Tween, and incubated with appropriate horseradish peroxidase (HRP)-conjugated secondary antibodies (Bio-Rad, Hercules, USA) for 1 hour at room temperature, before detection with Clarity western ECL substrate (Bio-Rad), following the manufacturer’s instructions. The chemiluminescence signal was detected by a ChemiDoc XRS+ with ImageLab software, and the densitometric analysis was performed using Image Lab software (all Bio-Rad). The relative band abundance was normalized versus ACT-β or vinculin, as the loading control. All the original western blots of all figures are available as supplementary material.

### Adenoviral infection

Recombinant adenovirus vectors were kindly donated by Junichi Sadoshima (Rutgers University) [82, 83]. For routine amplification of the vectors, HEK293 cells at 60% confluency were transduced with diluted supernatant from previous amplification rounds (1:4 ratio with fresh media); cells were collected 48 hours later by pipetting, and centrifuged at 300rcf for 10 minutes. The pellet was then resuspended in 1 ml PBS, and subjected to four cycles of sudden freezing in liquid nitrogen and thawing at 37 °C. Finally, the solution was centrifuged at 3250rcf for 30 minutes, the supernatant was collected and stored at −80 °C until use. For the ATG7 upregulation experiments, CSCs (1x10^5^ cells) were infected with a recombinant Adenovirus carrying ATG7 (Ad-ATG7) 1 day after plating. An adenovirus carrying the β-galactosidase gene (Ad-LacZ) was used as control in all overexpression experiments. Transduction efficiency was evaluated through ATG7 mRNA expression and protein level (Supplementary Fig. 3).

### mRFP-GFP-LC3 fluorescent dots assay

CSCs cultured in chamber-slides were transduced with Ad-mRFP-GFP-LC3 adenovirus for 48 hours [84]. After treatments, cells were washed with PBS, fixed with 4% paraformaldehyde, mounted with Vectashield (Vector Laboratories, Burlingame, USA), and viewed under a fluorescence Nikon Eclipse Ni microscope with VICO system (Nikon Corporation). The number of autophagosomes was determined by semiautomatic counting of GFP+/mRFP+ fluorescent puncta (yellow dots) under a 20X objective with the NIS Elements software (Nikon Corporation), normalizing to the cell surface area. At least 30 cells were analyzed for each condition; 30 pictures were taken for each sample.

### Small interfering RNA silencing

For ATG7 mRNA downregulation experiments, CSCs (1x10^5^ cells) were transfected with 100 µM of siRNA-ATG7 Smart pool (Dharmacon, Lafayette, USA) using Lipofectamine 2000 (Thermo-Fisher) in OptiMem (Thermo-Fisher), following the manufacturer’s instructions. A scramble (Scr) siRNA (Dharmacon) was used as control in all silencing experiments. Transfection efficiency was evaluated through ATG7 mRNA expression and protein level (Supplementary Fig. 4).

### Flow cytometry and apoptosis analysis

CSC immunophenotype was assessed by flow cytometry. Semi-confluent cultures were harvested with Accutase (Sigma-Aldrich) and stained with DDR2-APC (sc-81707, Santa Cruz), CD45-PerCP-cy5.5 (103131), Flk1-PE (121905), CD90-FITC (105305), Sca1-APC-cy7 (108125) antibodies (all Biolegend, San Diego, USA), according to the manufacturer’s guidelines. The percentage of apoptotic cells was measured by 7AAD/AnnexinV labelling (640908, BD Biosciences, San Jose, USA), according to the manufacturer’s modified protocol for adherent cells. Data acquisition was performed on a FACS-Aria II platform equipped with FACSDiva software (BD Biosciences), which was also used to calculate the compensation parameters. All flow cytometry data were analyzed with FlowJo software (FlowJo LLC, Ashland, USA), and plotted as fold change versus controls.

### RNA extraction and real-time PCR

Total RNA was extracted using column-based kits (Qiagen, Hilden, DE), according to the manufacturer’s instructions. RNA was quantified using NANODROP (Thermo-Fisher). For gene expression analyses, cDNA was synthesized from 0.5 μg of RNA with High Capacity cDNA Reverse Transcription Kit (Life Technologies, Carlsbad, USA). Real-time qPCR was performed using Power SYBR Green PCR Master Mix (Life Technologies) on a 7900HT Fast Real-Time platform (Applied Biosystems, Waltham, USA), using the manufacturer’s standard thermocycling conditions. The relative ratio versus control sample was calculated using the comparative Ct method (2^−ΔΔCt^), with ACT-β selected as the housekeeping gene, according to the Norm Finder algorithm (MOMA, Aarthus University, DK). ATG7 primers (FW= ATGATCCCTGTAACTTAGCCA, REV= CACGGAAGCAAACAACTT), ACT-β primers (FW= CACCAACTGGGACGACAT, REV= ACAGCCTGGATAGCAACG).

### Cytokine array for secretome profiling

Conditioned media were supplemented with 0.1% FBS and collected after the last 48 hours of culture from 3 independent experiments. Media were centrifuged at 2000rcf for 5 minutes to remove cells and debris, and then stored at −80 °C until analysis. Culture medium was assayed by the Proteome Profiler Mouse XL Cytokine Array (R&D Systems, Minneapolis, USA) to simultaneously detect 111 targets. Briefly, array membranes were blocked with blocking buffer for 1 hour at room temperature, and then 1 ml of culture medium was added to each membrane and incubated at 4 °C for overnight. Membranes were repeatedly washed, and then incubated with biotin-conjugated antibody for 1 hour at room temperature. After further washes, membranes were incubated for 30 minutes at room temperature with HRP-conjugated streptavidin, and washed one last time to remove unbound reagents. All incubation steps were performed under agitation on an orbital shaker. Membranes were then developed with the detection buffer following the manufacturer’s instructions. Briefly, arrays were scanned with ChemiDoc Imaging System (Bio-Rad), and spot signal densities were obtained using ImageLab software (Bio-Rad). The background was subtracted from the densitometry data, and the obtained values were normalized to the positive control spots for each membrane. A heatmap was generated using the log2-trasform densitometric quantification of each dot, using R package pheatmap (GNU Project). Euclidean distance was calculated using hclust clustering methods implemented in R software. Functional association network was created using the STRING database (ELIXIR Core Data Resources) selecting the “experiments” and “database” options.

### Angiogenesis assay

The angiogenesis assay was performed as previously described [29, 85]. Briefly, Matrigel matrix growth factor reduced (BD Corning, United States, Arizona) was seeded in a 96 multiwell plate and allowed to solidify for 1 hour at 37 °C. Human umbilical vein endothelial cells (HUVECs) were cultured in endothelial growth medium (EGM2, Lonza), seeded at a density of 2.5x10^4^ on top of the Matrigel layer, and cultured for 18 hours with CSC-conditioned media collected from the different treatments. As positive and negative controls, EGM-2 and DMEM 5 mm Glucose with 0.1%FBS were used, respectively. Images were taken under a fluorescence Nikon Eclipse Ti microscope (Nikon Corporation) with a 4X objective. Assay quantification, in terms of total tube length and number of loops, was performed with the Angiogenesis Analyzer Plugin of the ImageJ software (National Institutes of Health, Bethesda, MD).

### Statistical analysis

Statistical analysis was performed by GraphPad Prism 8 software (GraphPad Software, San Diego, USA). All results are presented as mean value ± standard error of the mean. Significance of difference between two groups was determined by two-sided Student’s *t*-test. When 3 or more groups were intercompared, parametric or non-parametric (as appropriate) one-way ANOVA test, followed by Bonferroni correction or uncorrected Dunn’s test for multiple comparisons, were used, respectively. A value of *P*<0.05 was considered to be significant.

## Supplementary information

Supplementary figures and tables

Full scan western blots

## Data Availability

The datasets generated and analyzed during the current study are available from the corresponding author on reasonable request.
