# Supplementary material for: The impact of autophagy modulation on phenotype and survival of cardiac stromal cells under metabolic stress
Source: Cell Death Discov. 2022 Apr 1;8:149. doi: 10.1038/s41420-022-00924-7 (PMC8975847; doi:10.1038/s41420-022-00924-7)
Supplement: Supplementary file 1 — Supplementary figures and tables [file 41420_2022_924_MOESM1_ESM.pdf]

SUPPLEMENTARY MATERIAL

The impact of autophagy modulation on phenotype and survival of cardiac stromal cells under metabolic stress

Isotta Chimenti* [1, 2 §]; Vittorio Picchio[1 §]; Francesca Pagano[3]; Leonardo Schirone[1, 4]; Sonia Schiavon[1], Luca D'Ambrosio[1], Valentina Valenti[5], Maurizio Forte[6], Flavio di Nonno[6], Speranza Rubattu[6,7], Mariangela Peruzzi[2], Francesco Versaci[5,8], Ernesto Greco[4], Antonella Calogero[1], Elena De Falco[1, 2], Giacomo Frati[1,6 §§]; Sebastiano Sciarretta[1,6 §§]

(1) Department of Medical Surgical Sciences and Biotechnologies, Sapienza University of Rome, Latina, Italy

(2) Mediterranea Cardiocentro, Napoli. Italy

(3) Biochemistry and Cellular Biology Istitute, CNR, Monterotondo, Italy

(4) Department of Clinical, Internal Medicine, Anaesthesiology and Cardiovascular Sciences, Sapienza University of Rome, Italy.

(5) Haemodynamic and Cardiology Unit, "Santa Maria Goretti" Hospital, Latina, Italy

(6) IRCCS Neuromed, Pozzilli, Italy

(7) Department of Clinical and Molecular Medicine, Sapienza University of Rome, Italy

(8) Department of System Medicine, "Tor Vergata" University, Rome, Italy

[§]These authors equally contributed to this work.

[§§]These authors are joint senior authors.

*Corresponding author: Isotta Chimenti, Corso della Repubblica 79, 04100 Latina, Italy. Email: isotta.chimenti@uniroma1.it. Phone: +3907731757234. Fax: +3907731757254.

SUPPLEMENTARY FIGURE 1

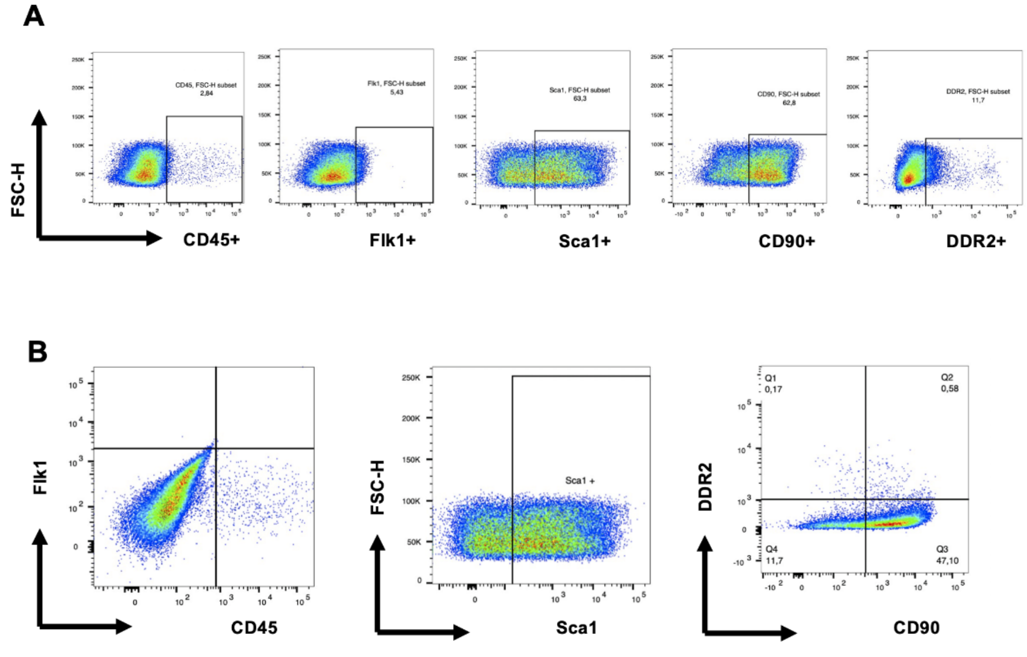

Supplementary figure 1 – Representative density plots of the flow cytometry analysis.

Representative panels are shown **(A)** for the quantification of the percentage of CD45+, Flk1+, Sca1+, CD90+, or DDR2+ single positive cells in the CSC population. Also, representative panels are displayed in **(B)** to show the gating strategy for the quantification of double positive DDR2/CD90 cells inside the Sca1+ population.

SUPPLEMENTARY FIGURE 2

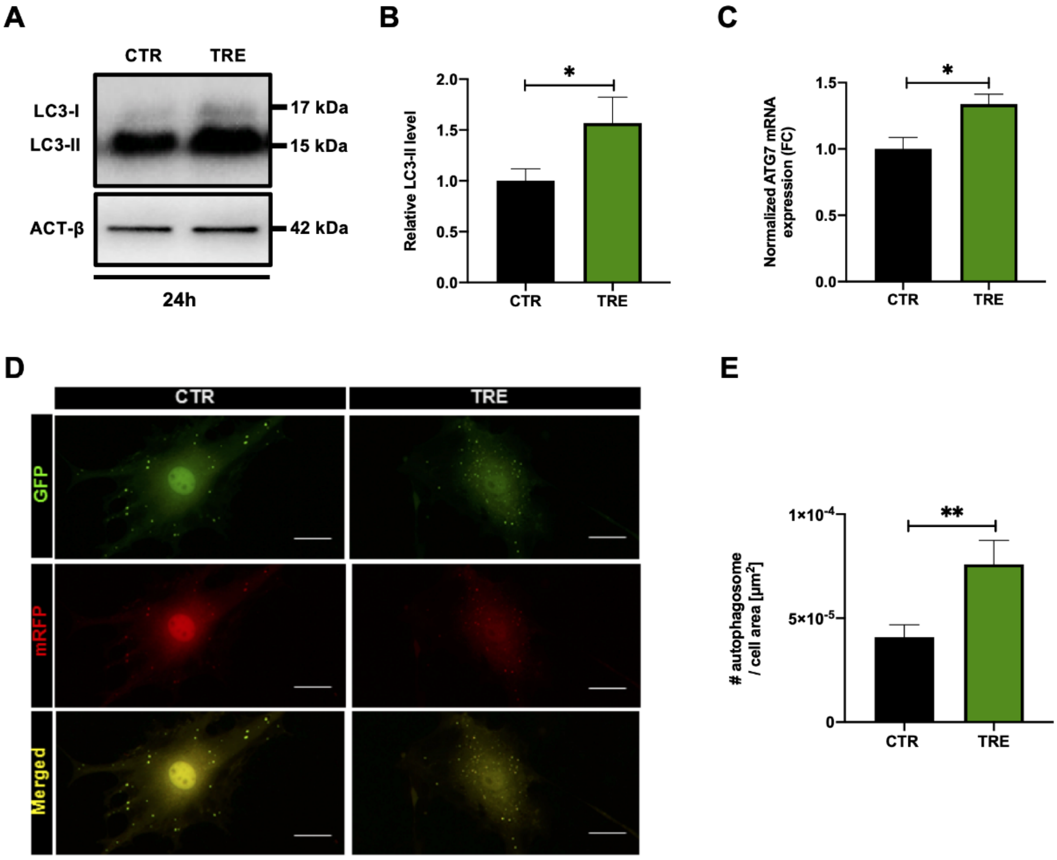

Supplementary figure 2 – Validation of in vitro autophagy modulation by trehalose in CSCs.

(A) Representative WB panels **(B)** and relative densitometric quantification of LC3-II protein expression profile in CSCs after 24 hours of trehalose (TRE) treatment (n=8). **(C)** ATG7 mRNA expression levels in CSCs after 24 hours of trehalose (TRE) treatment compared to control (CTR) (n=6). **(D)** Representative fluorescence microscopy images of TRE-treated or control CSCs, transduced with Ad-mRCF-GFP-LC3, and corresponding quantification of the number of mRCF+/GFP+ autophagosomes per cell surface area **(E)**. Scale bars=20μm (n=3). *P<0.05, **P<0.01.

SUPPLEMENTARY FIGURE 3

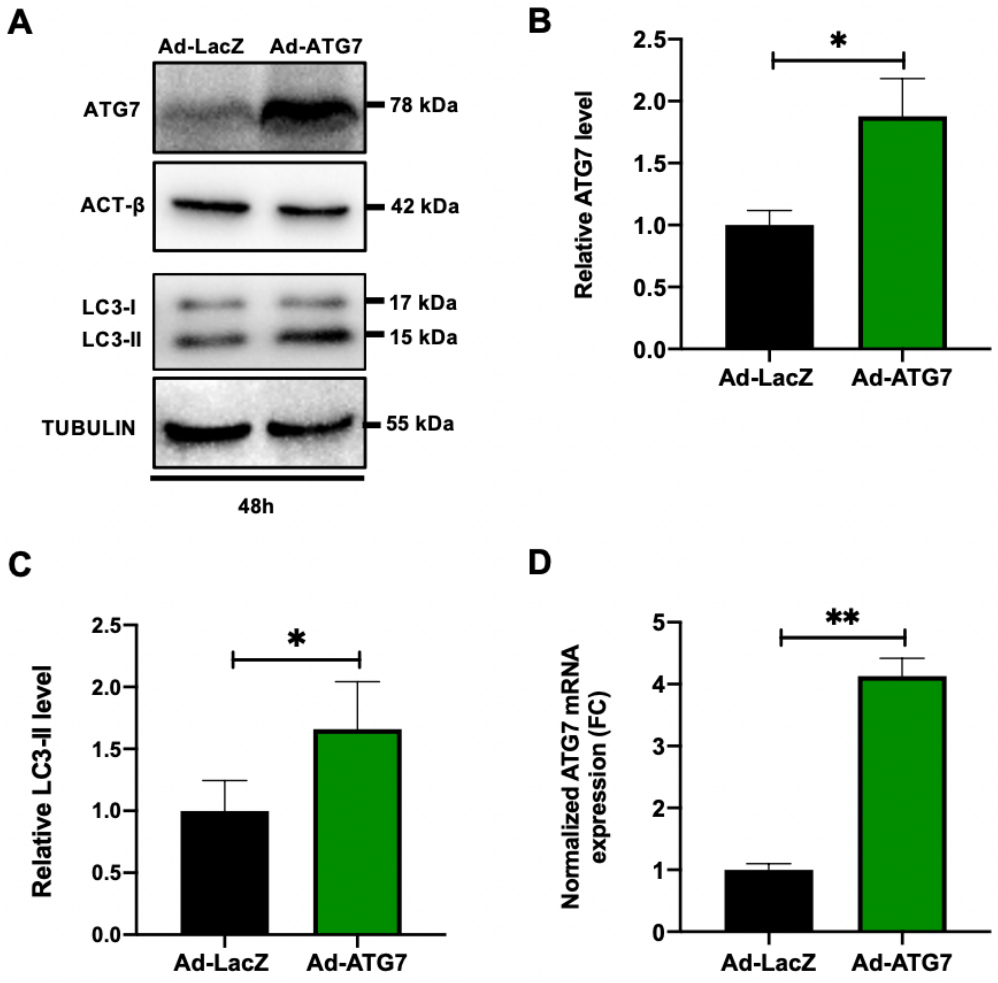

Supplementary figure 3 – Validation of autophagy modulation by adenovirus transduction.

Representative WB panels of ATG7 and LC3-II proteins **(A),** and relative densitometric quantification of ATG7 **(B)** or LC3-II **(C)** levels, 48 hours after ad-ATG7 or ad-LacZ transduction (n=7). **(D)** ATG7 mRNA expression levels in CSCs 48 hours after ad-ATG7 or ad-LacZ transduction (n=3). *P<0.05, **P<0.01.

SUPPLEMENTARY FIGURE 4

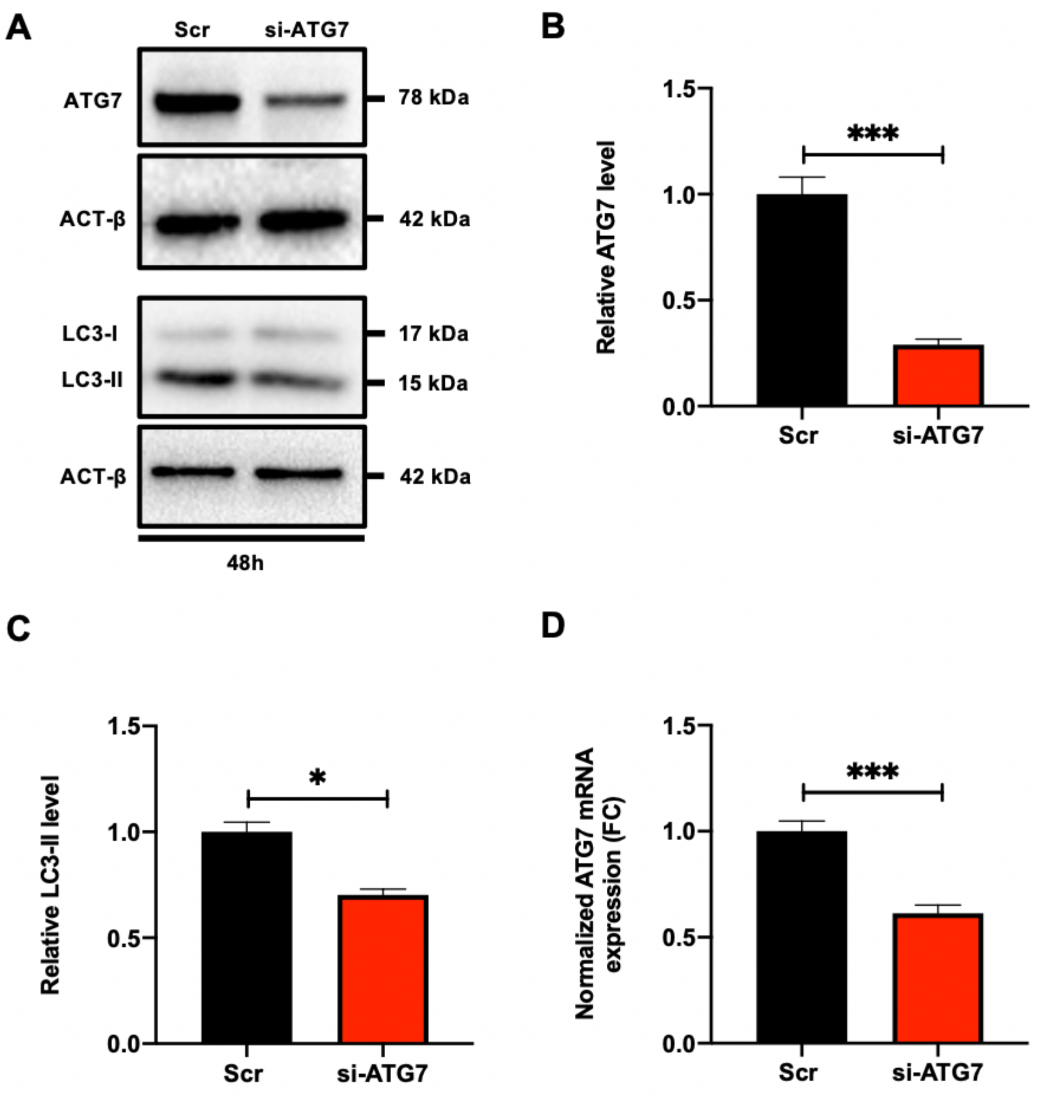

Supplementary figure 4 – Validation of autophagy modulation by RNA interference. Representative WB panels of ATG7 and LC3-II proteins **(A),** and relative densitometric quantification of ATG7 **(B)** or LC3-II **(C)** levels, 48 hours after transfection of a small interference RNA against ATG7 (si-ATG7) or scramble control (Scr) (n=4). **(D)** ATG7 mRNA expression levels in CSCs 48 hours after si-ATG7 or Scr transfection (n=3). *P<0.05, ***P<0.001.

SUPPLEMENTARY FIGURE 5

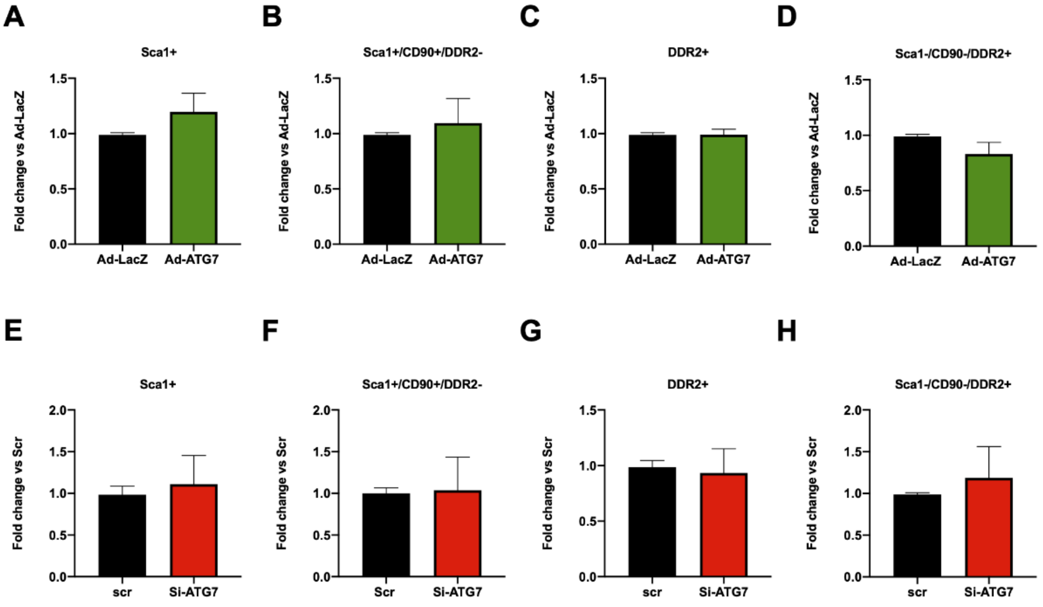

Supplementary figure 5 – Flow cytometry profile of CSCs after nutrient deprivation. Quantification of the Sca1+ **(A)**, Sca1+/CD90+/DDR2- **(B)**, DDR2+ **(C)**, and Sca1-/CD90-/DDR2+ **(D)** subpopulations after 32 hours of nutrient deprivation in Ad-ATG7 transduced CSCs. Corresponding histograms for the same subpopulations are also shown for si-ATG7 transfected CSCs (E-H, respectively) (n=8).

SUPPLEMENTARY FIGURE 6

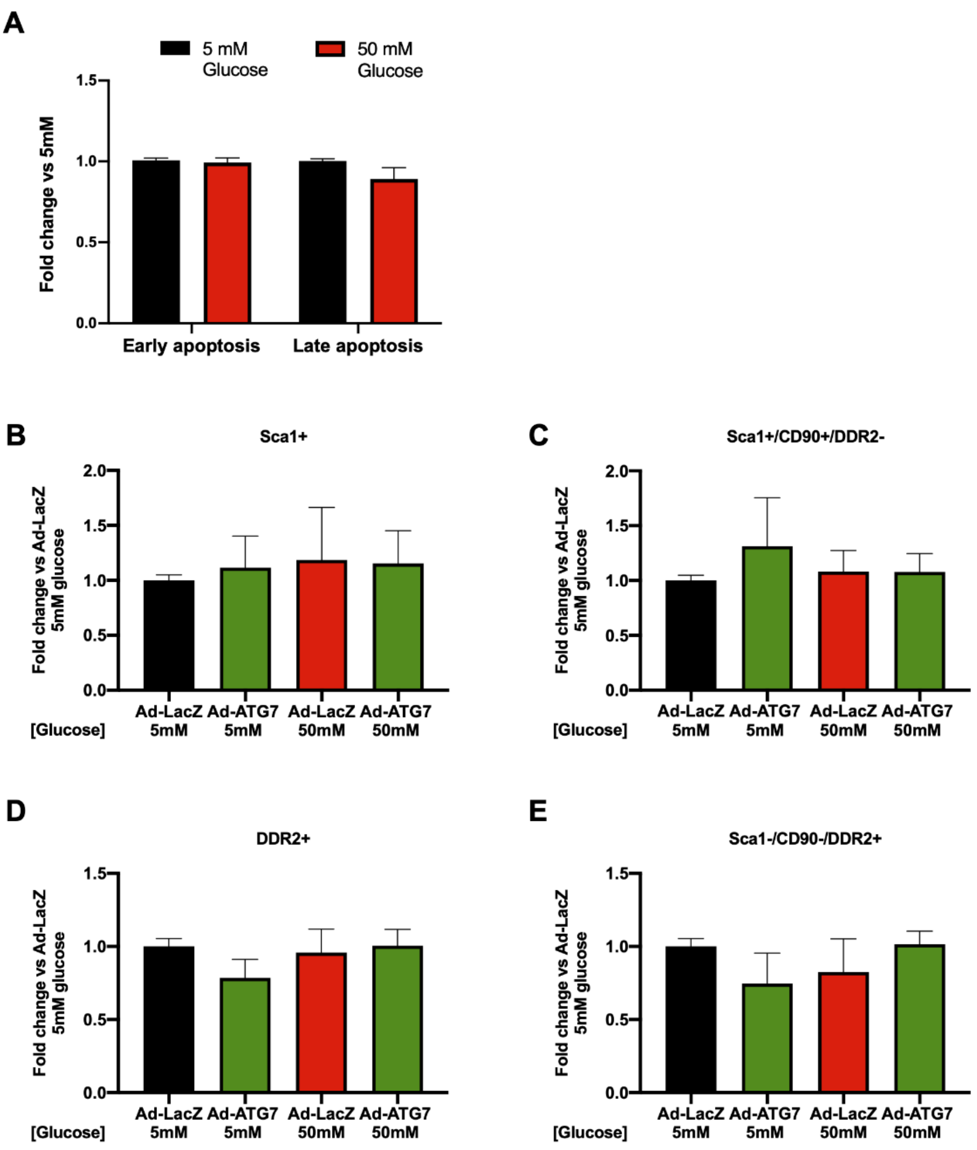

Supplementary figure 6 – CSC viability and immunophenotype after 1 week of hyperglycemia, with or without autophagy induction.

Quantification by flow cytometry of Annexin V/7AAD labelled CSCs after 1 week of culture in 5 or 50mM glucose **(A)**, with intermediate transduction with either Ad-Lacz or Ad-ATG7. Quantification of the Sca1+ **(B)**, Sca1+/CD90+/DDR2- **(C)**, DDR2+ **(D)**, and Sca1-/CD90-/DDR2+ **(E)** subpopulations in the same conditions (n=3).

SUPPLEMENTARY FIGURE 7

A

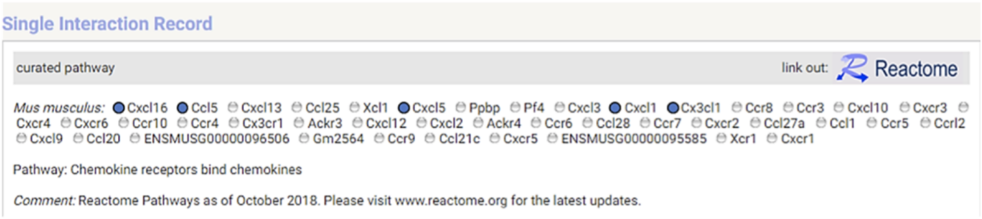

B

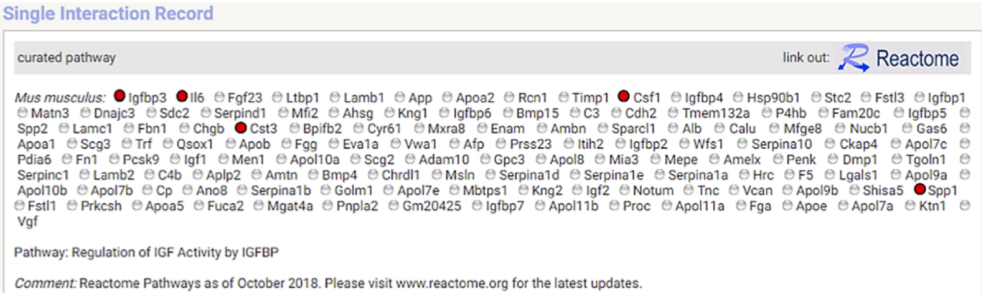

Supplementary figure 7 – Reactome pathways of interest.

Representative screenshots from the STRING database server of the cytokines included in the “Chemokine receptors bind chemokines” **(A)** and “Regulation of IGF activity by IGFBP” **(B)** pathways from REACTOME. The cytokines from our network of interest are highlighted by coloured bullets.

Supplementry Table 1. Gene Ontology (GO) with statistical analysis of downregulated cytokines. Selected GO-terms with the highest strength values from the functional network analysis on the STRING database.

**GO-term Biological Process**	**Description**	**Count in network**	**Strength**	**False Discovery Rate**
GO:0035684	helper T cell extravasation	2 of 2	3.06	4.68e-05
GO:0070949	regulation of neutrophil mediated killing of symbiont cell	2 of 4	2.76	0.00011
GO:0060754	positive regulation of mast cell chemotaxis	2 of 7	2.52	0.00022
GO:0036006	cellular response to macrophage colony-stimulating factor	2 of 8	2.46	0.00027
**GO-term Molecular Function**	**Description**	**Count in network**	**Strength**	**False Discovery Rate**
GO:0005041	low-density lipoprotein particle receptor activity	2 of 10	2.37	0.00030

GO:0045236	CXCR chemokine receptor binding	2 of 12	2.29	0.00039
GO:0008009	chemokine activity	6 of 41	2.23	4.20e-11
GO:0048020	CCR chemokine receptor binding	3 of 35	2.0	4.80e-05
GO:0005520	insulin-like growth factor binding	2 of 26	1.95	0.0013
**GO Term Cellular Component**	**Description**	**Count in network**	**Strength**	**False Discovery Rate**
GO:0005604	basement membrane	2 of 99	1.37	0.0403
GO:0031012	extracellular matrix	4 of 288	1.21	0.0031
GO:0005615	extracellular space	13 of 1131	1.13	2.30e-11
GO:0009897	external side of plasma membrane	3 of 308	1.05	0.0403
GO:0005576	extracellular region	16 of 2044	0.96	2.88e-12
