# Supplementary material for: The impact of autophagy modulation on phenotype and survival of cardiac stromal cells under metabolic stress
Source: Cell Death Discov. 2022 Apr 1;8:149. doi: 10.1038/s41420-022-00924-7 (PMC8975847; doi:10.1038/s41420-022-00924-7)
Supplement: Supplementary file 2 — Full scan western blots [file 41420_2022_924_MOESM2_ESM.pdf]

SUPPLEMENTARY MATERIAL

ORIGINAL WESTERN BLOT FULL SCAN

The impact of autophagy modulation on phenotype and survival of cardiac stromal cells under metabolic stress

Isotta Chimenti* [1, 2 §]; Vittorio Picchio[1 §]; Francesca Pagano[3]; Leonardo Schirone[1, 4]; Sonia Schiavon[1], Luca D'Ambrosio[1], Valentina Valenti[5], Maurizio Forte[6], Flavio di Nonno[6], Speranza Rubattu[6,7], Mariangela Peruzzi[2], Francesco Versaci[5,8], Ernesto Greco[4], Antonella Calogero[1], Elena De Falco[1, 2], Giacomo Frati[1,6 §§]; Sebastiano Sciarretta[1,6 §§]

(1) Department of Medical Surgical Sciences and Biotechnologies, Sapienza University of Rome, Latina, Italy

(2) Mediterranea Cardiocentro, Napoli. Italy

(3) Biochemistry and Cellular Biology Istitute, CNR, Monterotondo, Italy

(4) Department of Clinical, Internal Medicine, Anaesthesiology and Cardiovascular Sciences, Sapienza University of Rome, Italy.

(5) Haemodynamic and Cardiology Unit, "Santa Maria Goretti" Hospital, Latina, Italy

(6) IRCCS Neuromed, Pozzilli, Italy

(7) Department of Clinical and Molecular Medicine, Sapienza University of Rome, Italy

(8) Department of System Medicine, "Tor Vergata" University, Rome, Italy

[§]These authors equally contributed to this work.

[§§]These authors are joint senior authors.

*Corresponding author: Isotta Chimenti, Corso della Repubblica 79, 04100 Latina, Italy. Email: isotta.chimenti@uniroma1.it.

Phone: +3907731757234. Fax: +3907731757254.

FIGURE 2 A

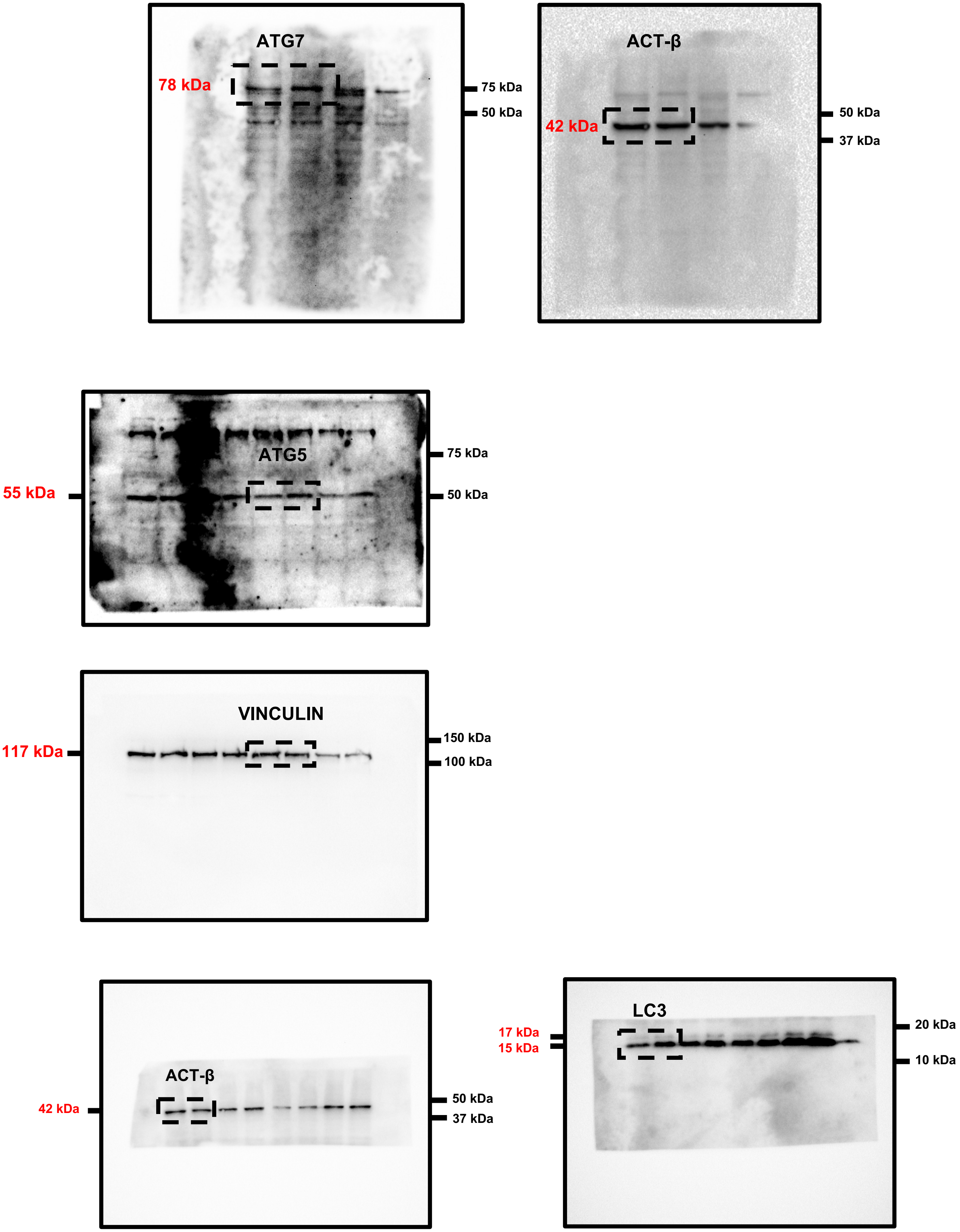

ATG7
78 kDa
75 kDa
50 kDa
ACT-β
42 kDa
50 kDa
37 kDa
ATG5
55 kDa
75 kDa
50 kDa
VINCULIN
117 kDa
150 kDa
100 kDa
ACT-β
42 kDa
50 kDa
37 kDa
LC3
17 kDa
15 kDa
20 kDa
10 kDa

FIGURE 2 D

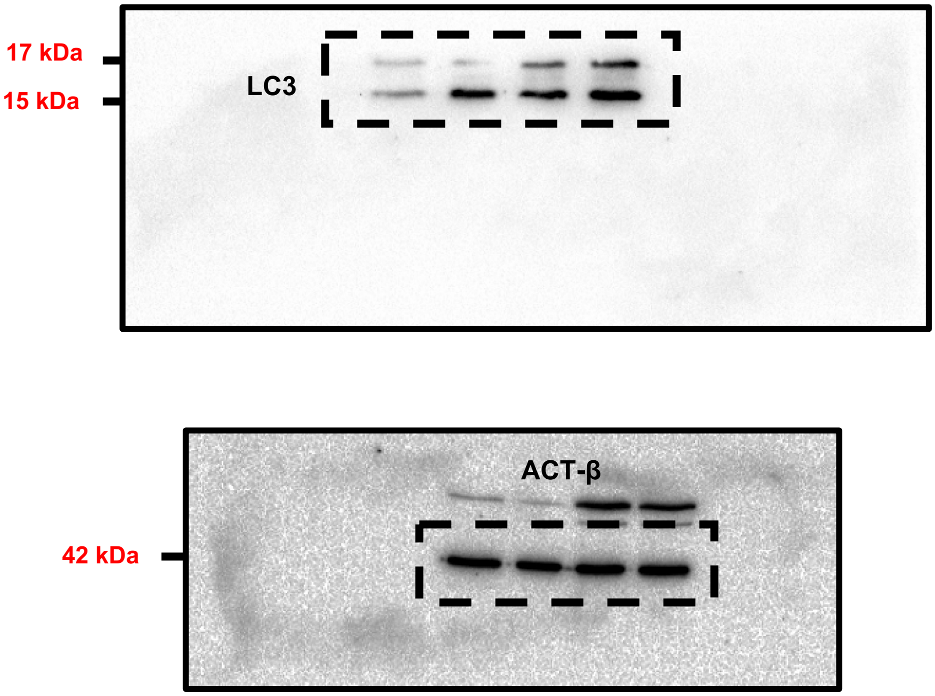

FIGURE 3

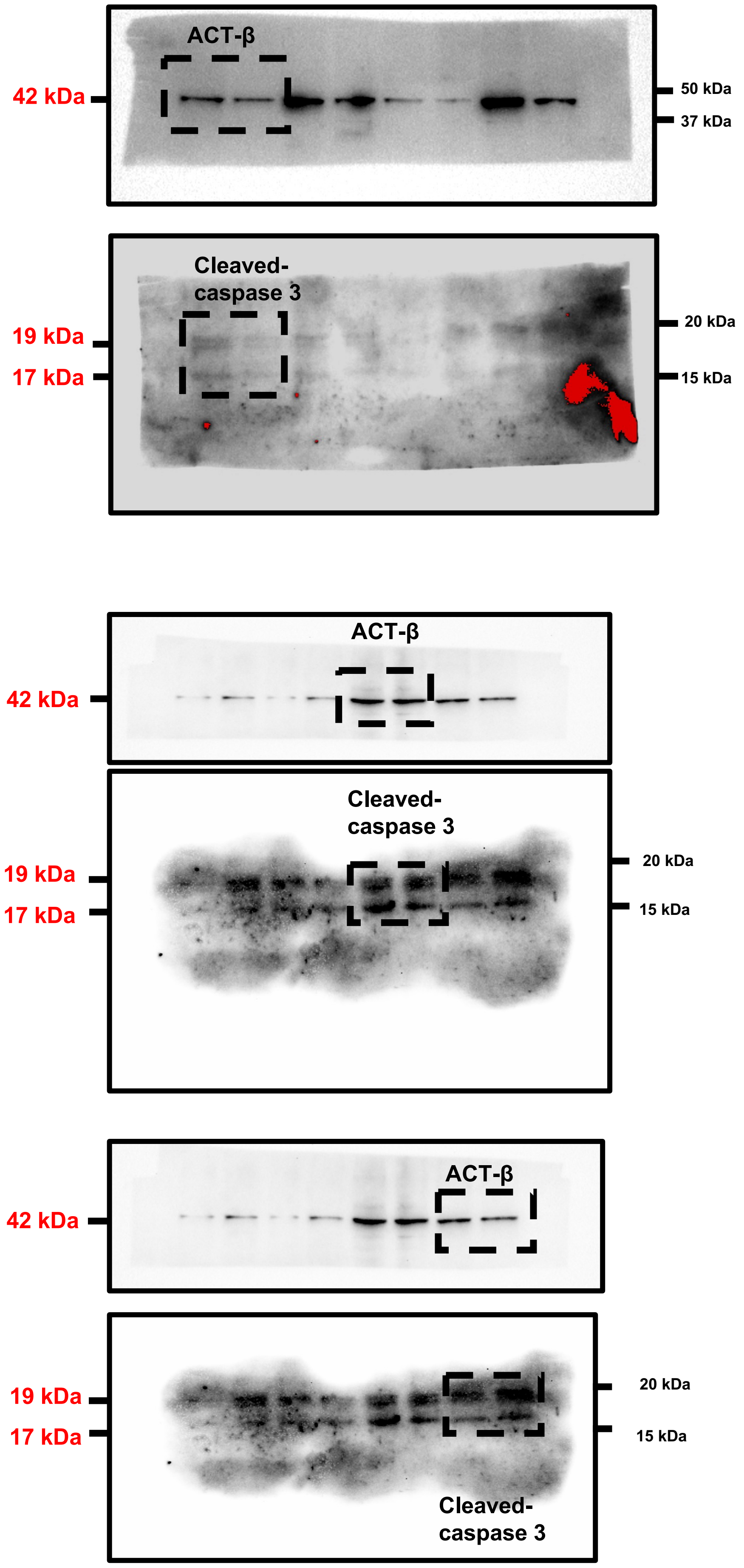
ACT-β
42 kDa
50 kDa
37 kDa
Cleaved-
caspase 3
19 kDa
17 kDa
20 kDa
15 kDa
ACT-β
42 kDa
Cleaved-
caspase 3
19 kDa
17 kDa
20 kDa
15 kDa
ACT-β
42 kDa
19 kDa
17 kDa
20 kDa
15 kDa
Cleaved-
caspase 3

FIGURE 5A

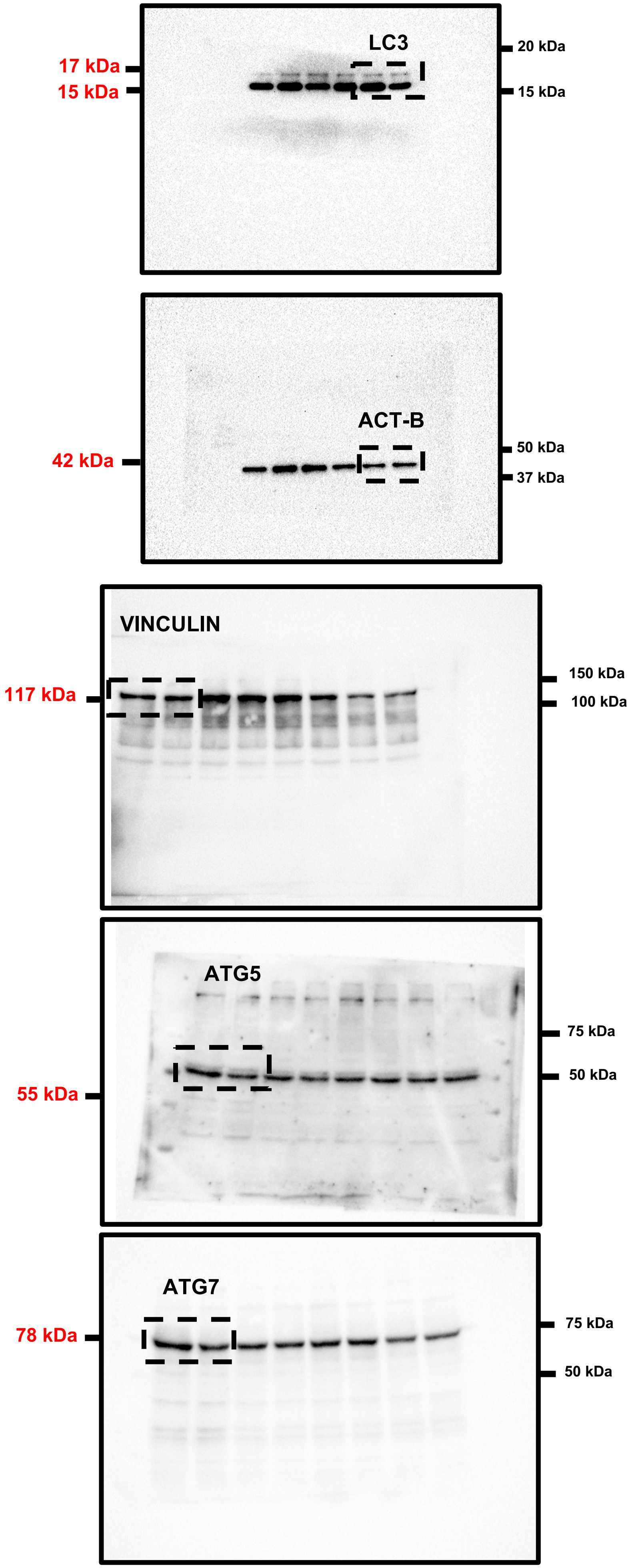

LC3
17 kDa
15 kDa
20 kDa
15 kDa
ACT-B
42 kDa
50 kDa
37 kDa
VINCULIN
117 kDa
150 kDa
100 kDa
ATG5
55 kDa
75 kDa
50 kDa
ATG7
78 kDa
75 kDa
50 kDa

FIGURE 5D

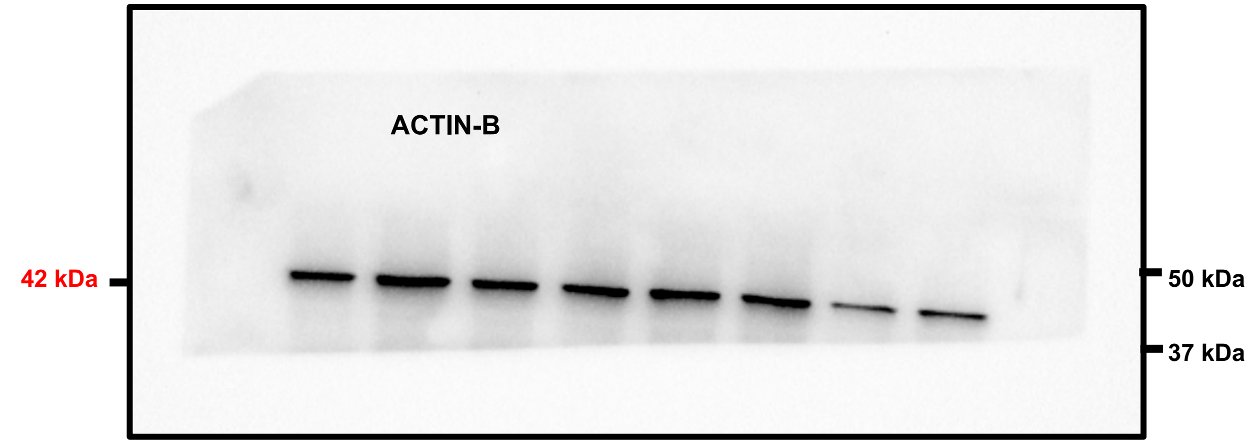

ACTIN-B
42 kDa
50 kDa
37 kDa

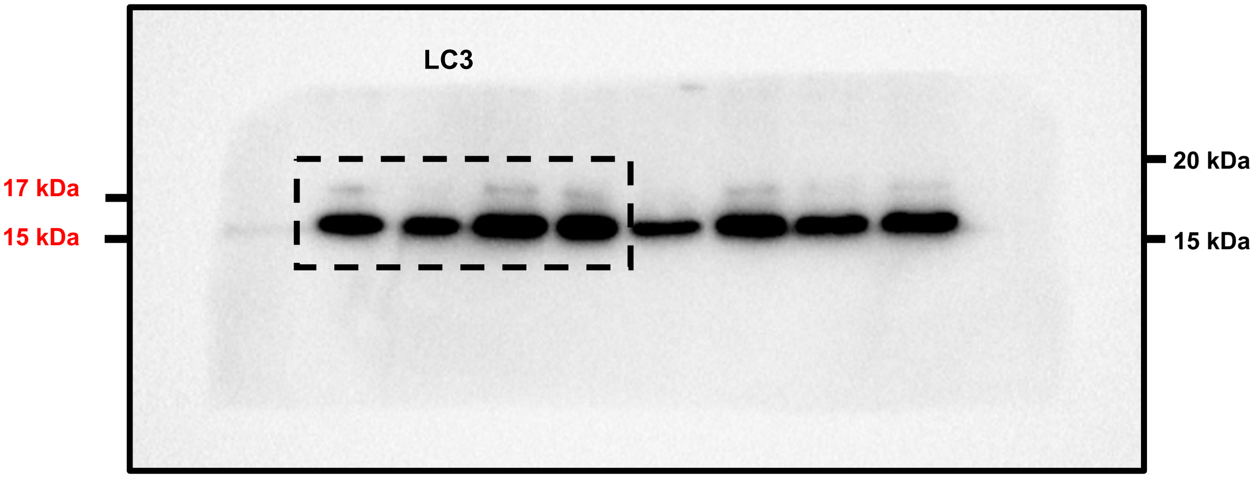

LC3
17 kDa
15 kDa
20 kDa
15 kDa

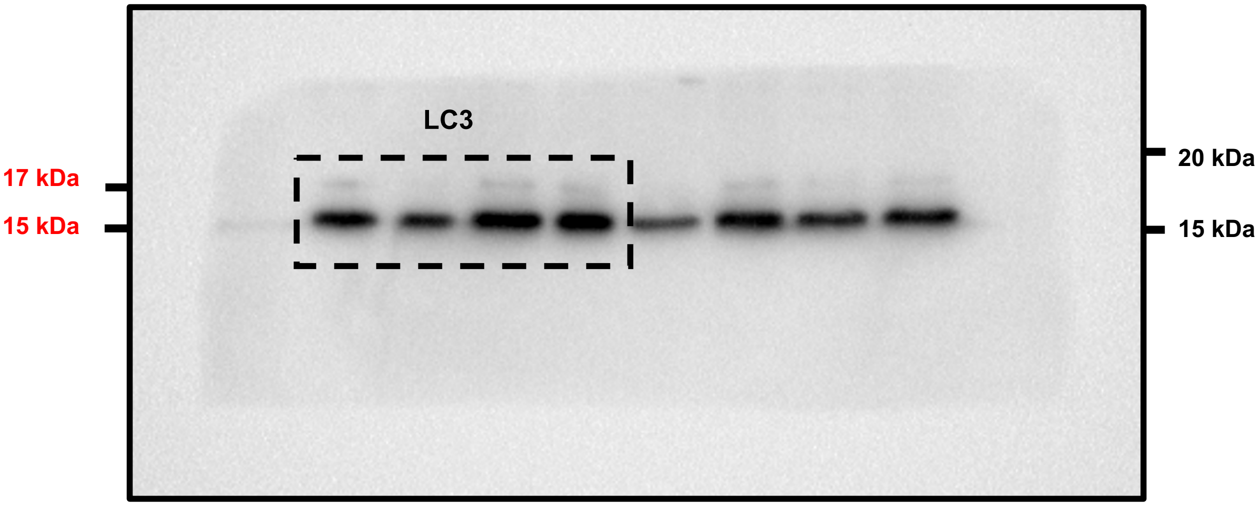

LC3
17 kDa
15 kDa
20 kDa
15 kDa

FIGURE SUP2

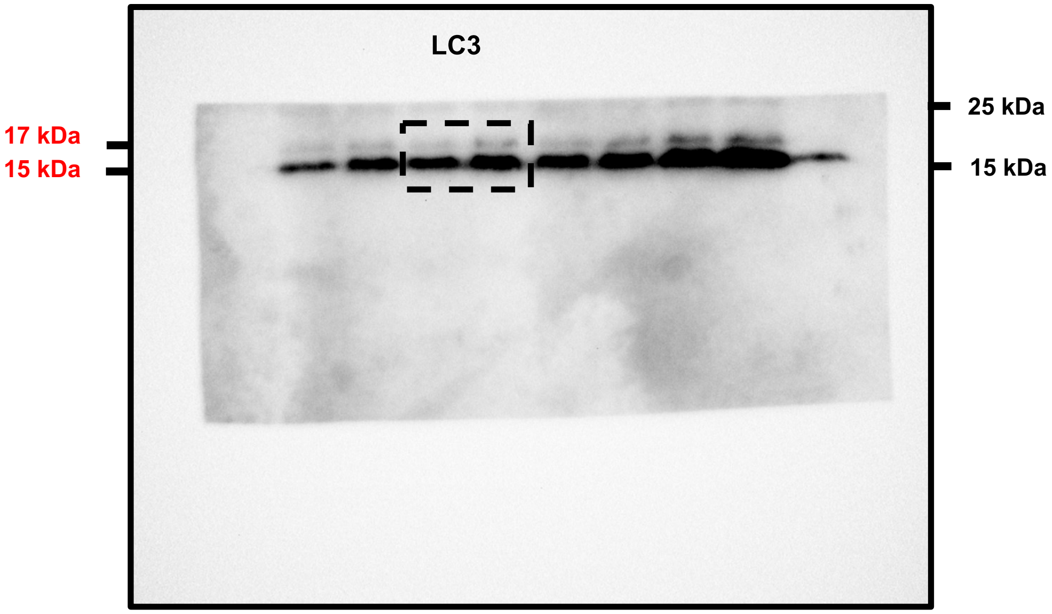

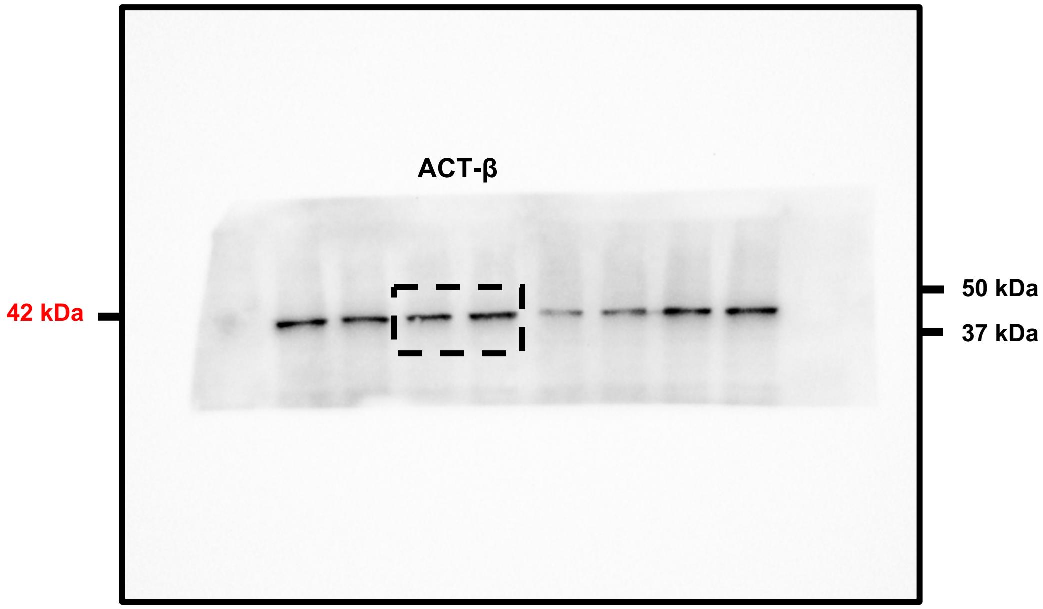

FIGURE SUP3

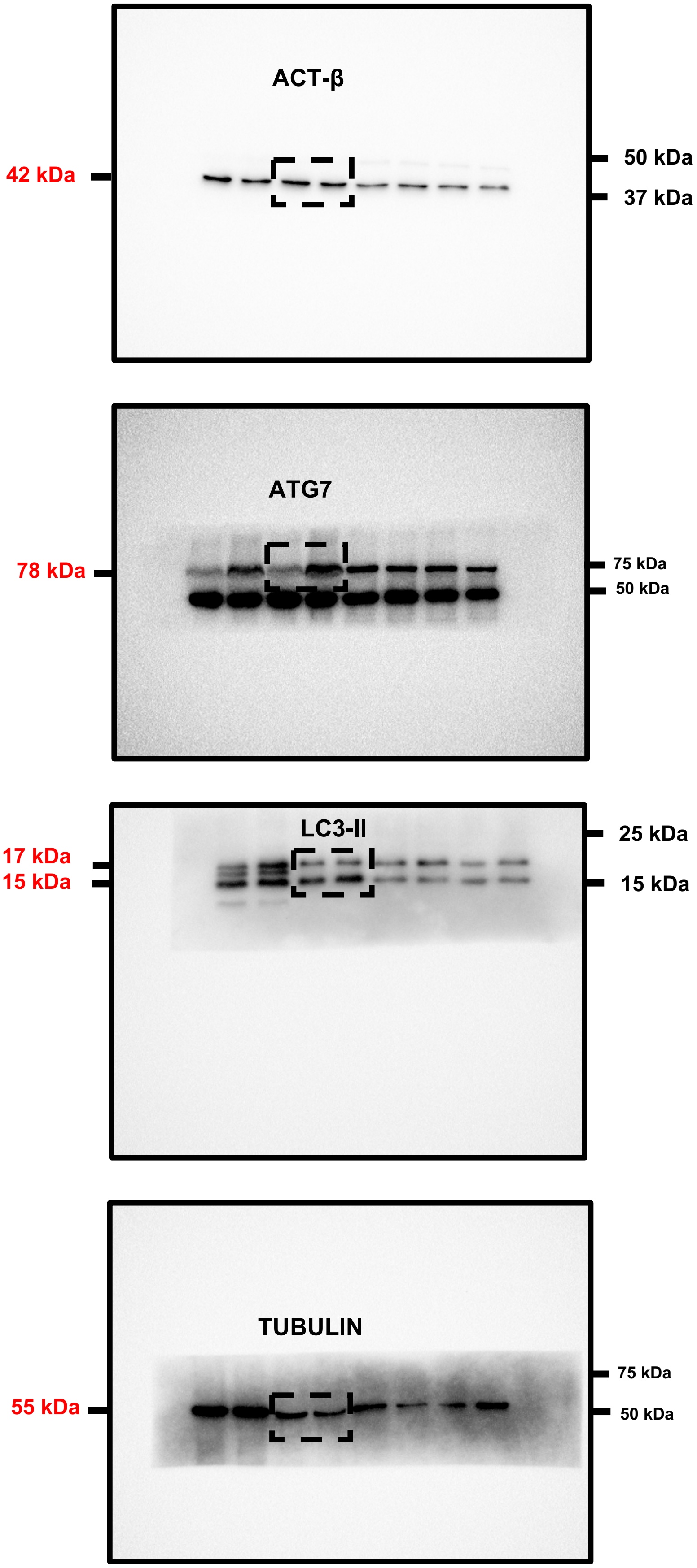

ACT-β
42 kDa
50 kDa
37 kDa
ATG7
78 kDa
75 kDa
50 kDa
LC3-II
17 kDa
15 kDa
25 kDa
15 kDa
TUBULIN
55 kDa
75 kDa
50 kDa

FIGURE SUP4

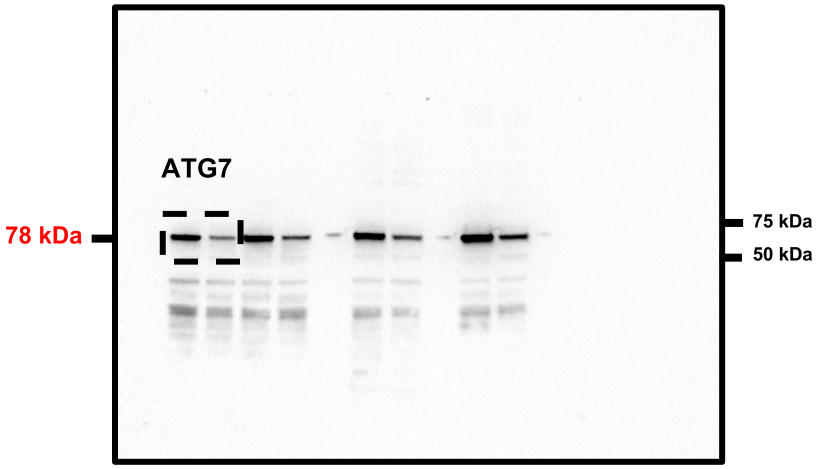

ATG7
78 kDa
75 kDa
50 kDa

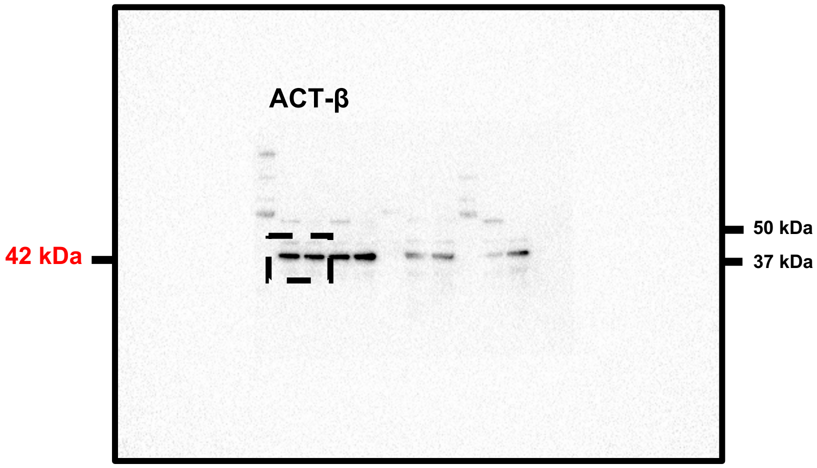

ACT-β
42 kDa
50 kDa
37 kDa

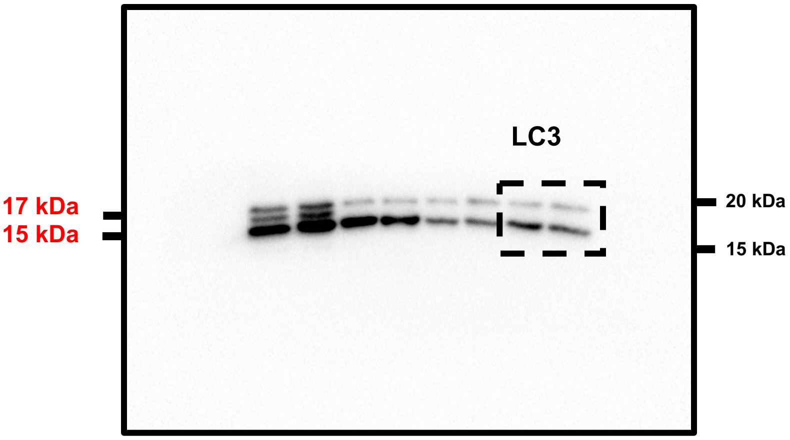

LC3
17 kDa
15 kDa
20 kDa
15 kDa

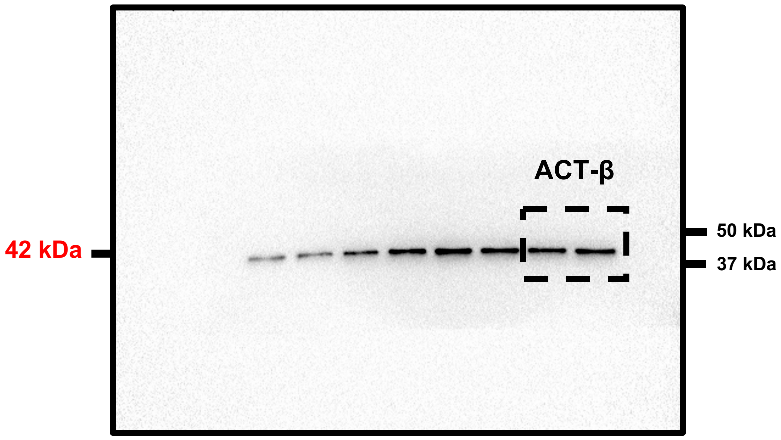

ACT-β
42 kDa
50 kDa
37 kDa
